# Mammalian cell growth dynamics in mitosis

**DOI:** 10.7554/eLife.44700

**Published:** 2019-05-07

**Authors:** Teemu P Miettinen, Joon Ho Kang, Lucy F Yang, Scott R Manalis

**Affiliations:** 1 MRC Laboratory for Molecular Cell BiologyUniversity College LondonLondonUnited Kingdom; 2 Koch Institute for Integrative Cancer ResearchMassachusetts Institute of TechnologyCambridgeUnited States; 3 Department of PhysicsMassachusetts Institute of TechnologyCambridgeUnited States; 4 Department of Biological EngineeringMassachusetts Institute of TechnologyCambridgeUnited States; 5 Department of Mechanical EngineeringMassachusetts Institute of TechnologyCambridgeUnited States; Institute of Cancer Research ResearchUnited Kingdom; Fred Hutchinson Cancer Research CenterUnited States

**Keywords:** cell growth, mitosis, cytokinesis, protein synthesis, CDK1, 4E-BP1, Chicken, Human, Mouse

## Abstract

The extent and dynamics of animal cell biomass accumulation during mitosis are unknown, primarily because growth has not been quantified with sufficient precision and temporal resolution. Using the suspended microchannel resonator and protein synthesis assays, we quantify mass accumulation and translation rates between mitotic stages on a single-cell level. For various animal cell types, growth rates in prophase are commensurate with or higher than interphase growth rates. Growth is only stopped as cells approach metaphase-to-anaphase transition and growth resumes in late cytokinesis. Mitotic arrests stop growth independently of arresting mechanism. For mouse lymphoblast cells, growth in prophase is promoted by CDK1 through increased phosphorylation of 4E-BP1 and cap-dependent protein synthesis. Inhibition of CDK1-driven mitotic translation reduces daughter cell growth. Overall, our measurements counter the traditional dogma that growth during mitosis is negligible and provide insight into antimitotic cancer chemotherapies.

## Introduction

Animal cell growth, that is biomass accumulation (Lloyd, 2013), is classically viewed to take place during interphase. During mitosis and cytokinesis, when cells are assumed to prioritize their energy usage for executing cell division, growth is presumed to be minimal (reviewed in Kronja and Orr-Weaver, 2011; Pyronnet and Sonenberg, 2001; Salazar-Roa and Malumbres, 2017; Sivan and Elroy-Stein, 2008; White-Gilbertson et al., 2009). More specifically, mRNA synthesis is inhibited due to chromatin condensation and dissociation of transcription factors (Liang et al., 2015; Novais-Cruz et al., 2018; Parsons and Spencer, 1997; Prescott and Bender, 1962), and ribosomal RNA synthesis is blocked as the nucleolus disappears in prometaphase (Hernandez-Verdun, 2011). Protein synthesis has also been reported to be suppressed in cell populations enriched for mitosis (Bonneau and Sonenberg, 1987; Celis et al., 1990; Fan and Penman, 1970; Prescott and Bender, 1962; Pyronnet et al., 2001; Sivan et al., 2011; Sivan et al., 2007). Consistently, polysome and ribosome profiling studies have suggested that the translational efficiency of most mRNAs is reduced in mitosis (Park et al., 2016; Qin and Sarnow, 2004; Stumpf et al., 2013; Tanenbaum et al., 2015). Studies on individual components of the translational machinery, such as eukaryotic Translation Initiation Factor 4E (eIF4E), eIF4E Binding Protein 1 (4E-BP1), eukaryotic Translation Elongation Factor 2 (eEF2) and S6 Ribosomal Protein (S6RP) have also suggested reduced protein synthesis, especially cap-dependent translation initiation, in mitosis (Celis et al., 1990; Dobrikov et al., 2014; Pyronnet et al., 2001; Shah et al., 2003; Wilker et al., 2007). Furthermore, ribosomes disassociate from endoplasmic reticulum around metaphase, suggesting that translation may be limited in the middle of mitosis (Puhka et al., 2007).

However, this classical view that growth is inhibited during mitosis has recently been challenged. Parts of DNA remain accessible for transcription machinery and de novo transcription of genes involved in cell growth persists in mitosis (Chan et al., 2012; Chen et al., 2005; Liu et al., 2017; Palozola et al., 2017). Recent reports also suggest that protein synthesis may persist during mitosis (Coldwell et al., 2013; Shuda et al., 2015; Stonyte et al., 2018). Importantly, cyclin-dependent kinase 1 (CDK1), the key regulator of mitotic entry and progression (Diril et al., 2012; Gavet and Pines, 2010), phosphorylates and activates components of the protein synthesis machinery, including 4E-BP1 (Heesom et al., 2001; Jansova et al., 2017; Shuda et al., 2015), eEF2 kinase (Smith and Proud, 2008) and p70 S6 kinase (Papst et al., 1998), suggesting an activation of cap-dependent translation. In addition, cap-independent translation of many mRNAs remains active in mitosis (Cornelis et al., 2000; Marash et al., 2008; Pyronnet et al., 2000; Qin and Sarnow, 2004). It is therefore becoming evident that particular proteins, especially those required for completion of cell division and those critical for cell growth, are synthesized during mitosis (Aviner et al., 2013; Aviner et al., 2017; Cornelis et al., 2000; Marash et al., 2008; Park et al., 2016; Pyronnet et al., 2000; Stumpf et al., 2013; Tanenbaum et al., 2015). However, the extent and dynamics of protein synthesis in mitosis remain unclear.

Importantly, protein and RNA synthesis rates are only proxies of overall growth (biomass increase), which is determined by the balance between synthesis (anabolic) and degradation (catabolic) rates (Lloyd, 2013; Miettinen and Björklund, 2015; Miettinen et al., 2017). Overall growth behavior during mitosis has not been studied, primarily due to the lack of precise cell size measurement methods that are sensitive enough to quantify growth during the short mitotic stages. Here, we utilize suspended microchannel resonator (SMR), a high-precision microfluidic mass sensor, and protein synthesis assays in conjunction with cell cycle measurements to study the extent, dynamics, mechanisms and consequences of mitotic growth on a single-cell level.

## Results

### Animal cells grow during mitosis and cytokinesis

**Figure 1.**
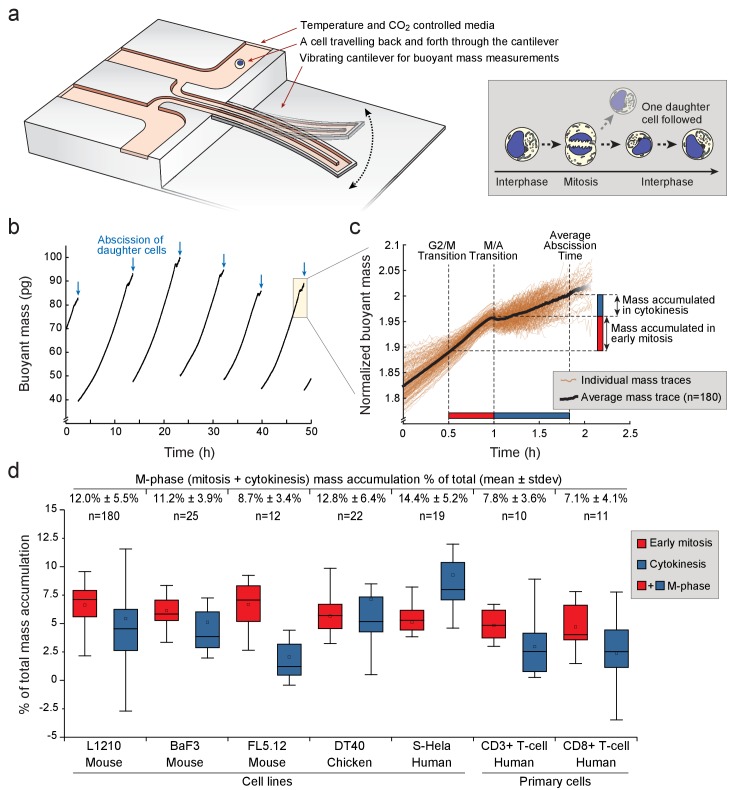
Various animal cell types grow during mitosis and cytokinesis. (**a**) *Left,* schematic of a suspended microchannel resonator (SMR). Single-cell buoyant mass is repeatedly measured as the cell flows back and forth through the vibrating cantilever. *Right,* at cell division, one of the daughter cells is randomly selected and monitored, while the other daughter cell is discarded from the SMR. (**b**) Buoyant mass trace of a single L1210 cell and its progeny over five full generations. The interdivision time (~9 hr) for cells growing in the SMR and in normal cell culture condition is equivalent. Blue arrows indicate the abscissions of daughter cells. (**c**) Overlay of 180 individual L1210 cell buoyant mass traces (transparent orange) and the average trace (black) around mitosis. Each mass trace has been normalized so that the typical cell abscission mass is 2. (**d**) Mass accumulation in mitosis (before metaphase/anaphase transition, red) and cytokinesis (blue) relative to the total mass accumulated during the cell cycle for various animal cell types Total relative mass accumulation in M-phase (sum of mitosis and cytokinesis) is indicated on top. Note that while the relative mass accumulation in cytokinesis varies between cell types, all cell types display similar mass accumulation % in early mitosis. n refers to the number of individual cells analyzed. Boxplot line: median, box: interquartile range, whiskers: ± 1.5 x interquartile range. 10.7554/eLife.44700.005Figure 1—source data 1.L1210 buoyant mass measurement data.

SMR is a microfluidic cantilever that is capable of measuring buoyant mass (a proxy of dry mass, referred to as mass from here on) of single cells with a precision of <0.1 pg (Figure 1a; Figure 1—figure supplement 1a–d) (Burg et al., 2007; Son et al., 2015a; Son et al., 2012). This resolution corresponds to <8 nm (<0.07%) change in a spherical lymphocyte cell diameter. We repeatedly measured mass of the same cell every ~1 min, resulting in a temporal resolution of approximately 2 min according to the Nyquist rate. We quantified growth, more specifically mass accumulation, throughout multiple cell cycles in L1210 mouse lymphocytes without perturbing normal growth rates (Figure 1b) (Son et al., 2015a; Son et al., 2012). We assigned approximate mitotic entry (i.e. G2/M transition), metaphase-to-anaphase transition (i.e. M/A transition) and cytokinetic abscission of the daughter cells for each cell using biophysical properties and FUCCI cell cycle reporter (Figure 1—figure supplement 2; Materials and methods) (Kang et al., 2019). This allowed quantification of mass accumulation during early mitosis (between G2/M transition and M/A transition) and cytokinesis (between M/A transition and daughter cell abscission) on a single-cell level (Figure 1c). In cytokinesis the elongated cells register smaller than round cells in our mass measurements, because of a change in mass distribution (Kang et al., 2019). Correcting for this cell elongation induced bias (correction is applied to all data shown, unless otherwise stated) (see Materials and methods), had little influence of the mass measurements during cytokinesis (Figure 1—figure supplement 1e).

**Figure 1—figure supplement 1.**
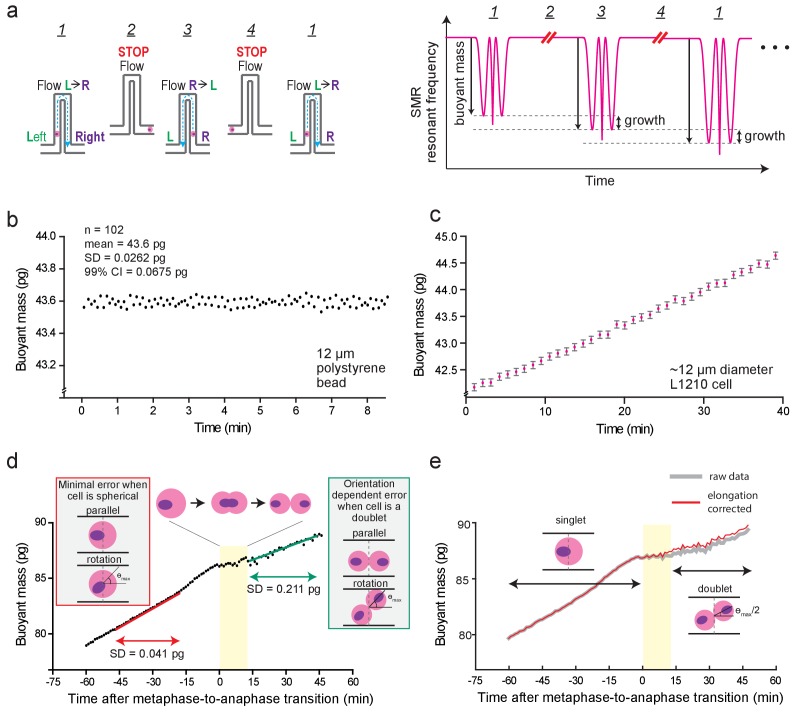
Suspended microchannel resonator (SMR) setups and noise characterization. (**a**) *Left,* schematic of automated fluid control strategy for continuous single-cell mass measurements. Steps in order: 1) A single cell (pink circle) flows left to right. Flow direction is depicted in blue dashed lines. 2) Once cell reaches right side of the cantilever, flow is stopped (~50 s). 3) Flow direction is reversed, and the cell flows to the left side. 4) Flow is stopped again (~50 s). These steps (1-4) are repeated to continuously measure the buoyant mass of the cell as it grows within the SMR. *Right,* schematic of SMR resonant frequency readout during steps depicted on left. Cell buoyant mass (i.e. height of the two side peaks) increases between each measurement, which corresponds to cell growth. (**b**) SMR measurement noise quantification by repeated buoyant mass measurements of a single 12 µm polystyrene bead. (n = 102 repeated measurements). (**c**) Representative 40 min buoyant mass trace of a L1210 cell (n = 180 individual cells). Pink dots depict each measurement and gray error bars depict the 99% confidence interval (CI) obtained from the repeated bead measurement shown in (**b**). (**d**) Orientation-dependent noise in mass measurements. Representative buoyant mass trace of a L1210 near mitosis is shown (n = 180 individual cells). Before anaphase L1210 cells are highly spherical and orientation-dependent noise is minimal (left inset, red box). The SD is comparable to the noise obtained from repeated bead measurement. After cell elongation (singlet to doublet), noise increases due to orientation-dependent error (right inset, green box). See Materials and methods for additional details. (**e**) Cell elongation induced buoyant mass measurement bias in cytokinesis. Representative buoyant mass trace of a L1210 near mitosis is shown with (red) and without (grey) the cell elongation correction in data analysis (n = 180 individual cells). The yellow area represents the duration of cell elongation, as in panel (**d**). See Materials and methods for additional details.

**Figure 1—figure supplement 2.**
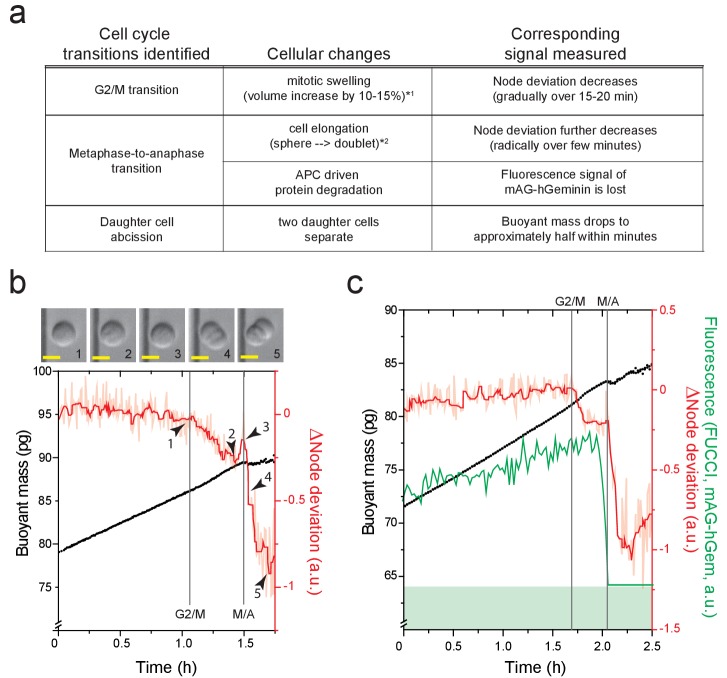
Detection of cell cycle transitions. (**a**) A table summarizing cellular changes and corresponding signals measured in SMR. (*one refers to Son et al., 2015a; *two refers to Kang et al., 2019). Node deviation is an acoustics-based measurement that depends on cell shape and stiffness (Kang et al., 2019). (**b**) Example buoyant mass (black) and node deviation (raw: light red, filtered: red) traces of a L1210 during G2 and M-phase (n = 180 individual cells). ∆Node deviation represents a change in node deviation compared to median of first 15 data points. Node deviation decreases as a cell enters mitosis (arrowhead #1) due to cell swelling (Kang et al., 2019), which starts immediately after mitotic entry (Son et al., 2015a; Zlotek-Zlotkiewicz et al., 2015). This is followed by another rapid decrease in node deviation after metaphase-to-anaphase transition (arrowheads #3 and #4). Vertical lines mark G2/M and metaphase-to-anaphase (M/A) transition, respectively. A cell morphology acquired by on-chip imaging at indicated time points (arrow heads and numbers) are shown on top. Scale bars denote 10 µm. (**c**) Example buoyant mass (black), node deviation (raw: light red, filtered: dark red) and FUCCI (green, mAG-hGem) traces of a L1210 FUCCI cell during G2 and M-phase (n = 12 individual cells). Fluorescence detection limit of our system is shown by a green band in the bottom. An abrupt loss of FUCCI signal (degradation of mAG-hGem) marks the metaphase-to-anaphase transition.

In total, we analyzed 180 individual L1210 cells undergoing mitosis and observed that on average 12% of the total mass accumulated during the whole cell cycle was acquired during M-phase (i.e. during mitosis and cytokinesis) (Figure 1d; Figure 1—source data 1). 7% of total cell growth took place during early mitosis, while 5% took place during cytokinesis. During anaphase, duration of which was estimated based on cell elongation (Materials and methods), mass accumulation was negligible. Considering that in most cell lines M-phase lasts approximately 10% of the whole cell cycle, the 12% mass accumulation observed during M-phase makes M-phase growth comparable to interphase growth.

The extent of total cell growth during mitosis and cytokinesis was surprising. To determine how generalizable this finding is, we repeated our measurements in other animal cell types. Mouse FL5.12 and BaF3 pro-B lymphocytes, and chicken DT40 lymphoblasts grew 9–13% of their total mass in M-phase (Figure 1d). Suspension HeLa (S-HeLa) cells also grew 14% of their total mass during M-phase, validating that substantial growth in M-phase is not specific to lymphocytes. We also examined CD3 +and CD8+activated primary human T cells. Both T-cell subpopulations added approximately 7% of their total mass during M-phase. Thus, growth in mitosis and cytokinesis is an important contributor to the total cellular growth across a variety of cell types grown in suspension.

### Cell mass accumulation persists through prophase, stops as cells approach metaphase-to-anaphase transition and recovers during late cytokinesis

**Figure 2.**
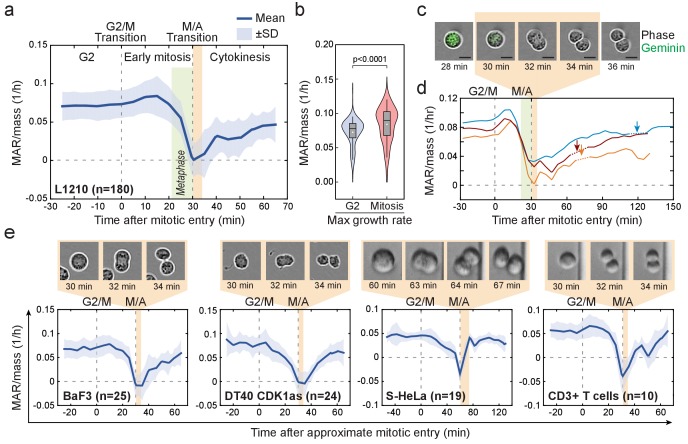
Cell mass accumulation persists through prophase, stops as cells approach metaphase-to-anaphase transition and recovers during late cytokinesis. (**a**) Mass-normalized mass accumulation rate (MAR) of L1210 cells in late G2 and M-phase. G2/M and metaphase-to-anaphase transitions are indicated with dashed vertical lines. Typical durations of metaphase and cell elongation (singlet to doublet) are indicated in green and light brown areas, respectively. n refers to number of individual cells analyzed. (**b**) Quantification of L1210 cell maximal growth rate in late G2 and in mitosis (n = 180 cells). p-Values obtained using two-tailed Welch’s t-test. In boxplots, line: median, box: interquartile range, whiskers: 5–95%. (**c**) Representative L1210 cell phase contrast (grey) and mAG-hGeminin cell cycle reporter (green) images (n = 18 cells). Times correspond to (**a**) and (**d**). Note that the physical separation of daughter cells takes place when cells are not accumulating mass. (**d**) Examples of individual L1210 mass-normalized MAR traces in late G2, M-phase and early G1. Arrows indicate the final abscission of daughter cells, around which mass-normalized MAR is indicated with dashed lines. M/A denotes the metaphase-to-anaphase transition, G2/M denotes the approximate mitotic entry, both of which are indicated with dashed vertical lines. (**e**) Mass-normalized MAR of indicated cell types along with representative images displaying the duration of the physical separation of daughter cells. BaF3 and DT40 cells were imaged separately, whereas S-HeLa and CD3 +T cells were imaged on-chip simultaneously with MAR measurements. M/A denotes the metaphase-to-anaphase transition, G2/M denotes the approximate mitotic entry, both of which are indicated with dashed vertical lines. Solid dark blue lines indicate the mean and light blue areas represent ± SD. n refers to number of individual cells analyzed.

To study the dynamics of cell growth during M-phase, we quantified the absolute mass accumulation rates (MAR) before and during M-phase (Figure 2—figure supplement 1; Materials and methods). To account for cell-size-dependent growth rates (Miettinen and Björklund, 2016; Miettinen et al., 2017; Son et al., 2012), we normalized MAR to the mass of the cell (MAR/mass). Surprisingly, after mitotic entry (during approximate prophase) L1210 cells exhibited on average 15.8 ± 3% (mean ± SEM, n = 180) increase in MAR when compared to late G2 phase (Figure 2a,b). As cells approached the metaphase-to-anaphase transition, MAR rapidly decreased and eventually reached zero at the end of metaphase. MAR remained near zero for the approximate duration of anaphase, after which MAR started to recover during late cytokinesis (Figure 2a,c,d). The recovery of MAR continued through the abscission of daughter cells (Figure 2d). These cell growth dynamics also persisted under different nutrient conditions and were reproducible with different SMR devices over multiple years of study (Figure 2—figure supplement 2a,c).

**Figure 2—figure supplement 1.**
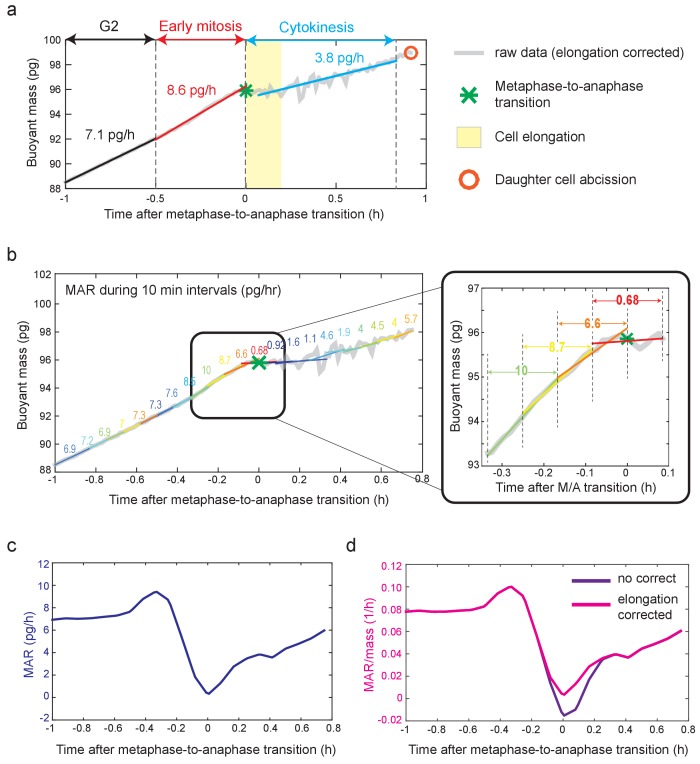
Mass accumulation rate (MAR) analysis. (**a**) Schematic of obtaining the average MAR/mass for late G2, early mitosis and cytokinesis. The following steps have been performed in order. First, metaphase-to-anaphase transition (green star) and approximate mitotic entry were specified based on biophysical and fluorescence markers (see Figure 1—figure supplement 2). Second, late G2, early mitosis and cytokinesis were selected as described in the Materials and methods. Briefly, for L1210 cells shown here, cytokinesis was set as a 50 min period after metaphase-to-anaphase transition; early mitosis was set as a 30 min period before metaphase-to-anaphase transition, and late G2 was set as 30 min period prior to the start of early mitosis. Vertical dashed lines separate each stage. Third, buoyant mass traces (grey) during each stage were linearly fitted and the slope of each line represents the MAR. Finally, MAR of each stage was divided by the average mass within that stage. Cell elongation period (12 min for L1210 cells) is depicted in yellow. Orange circle denotes daughter cell abscission. (**b**) Schematic of obtaining MAR with high-temporal resolution. The following steps has been performed in order. First, metaphase-to-anaphase transition (green star) was specified as described above. Second, a 10-min period around metaphase-to-anaphase is selected (i.e. ± 5 min around metaphase-to-anaphase). Third, the rest of the buoyant mass trace is divided in to additional 10 min periods, each shifted by 5 min. Fourth, each section is then fitted with a linear line, and the slope of each fitted line represents MAR at the center of that line. For example, 0.68, which represents the slope of 10 min period around metaphase-to-anaphase transition, represents MAR at t = 0 (see inset on right). Lastly, the MAR at each period is divided by the average mass of that 10 min period to obtain MAR/mass. Note that for Figure 5, similar analysis was carried out using 4 min periods, each shifted by 2 min. (**c**) MAR vs time trace that was obtained from data in panel (**b**) after smoothing of the buoyant mass trace. (**d**) MAR/mass vs time trace obtained from panel (**b**) after smoothing of the buoyant mass trace. MAR/mass trace of elongation corrected (magenta) and uncorrected (purple) are plotted together for comparison.

**Figure 2—figure supplement 2.**
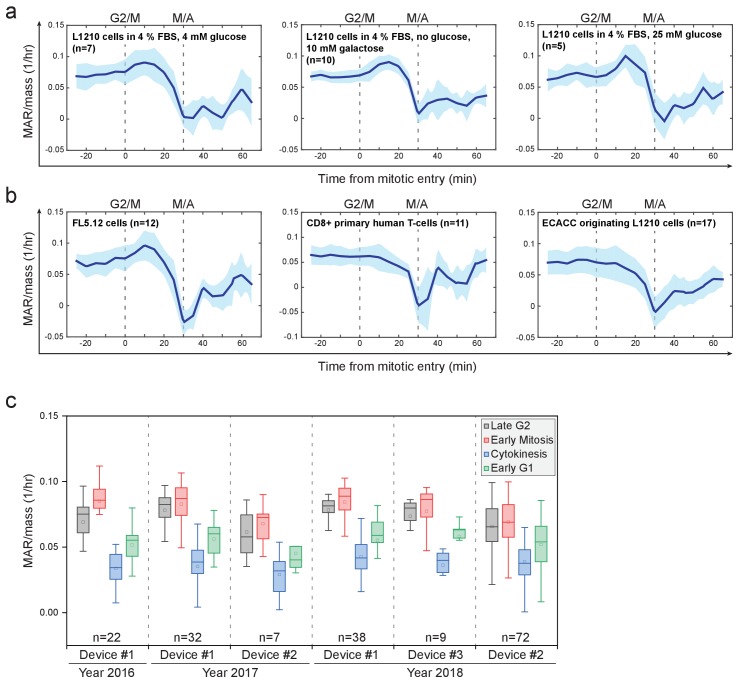
MAR in different growth conditions and cell types. (**a**) Mass-normalized MAR of L1210 cells in late G2 and M-phase when grown under indicated FBS and glucose conditions. For comparison, in Figure 2a (as well as all other figures), L1210 cells were grown in 10% FBS and 11 mM glucose conditions. M/A denotes the metaphase-to-anaphase transition, G2/M denotes the approximate mitotic entry, both of which are indicated with dashed vertical lines. Solid dark blue lines indicate the mean and light blue areas represent ± SD. n refers to number of individual cells analyzed. (**b**) Mass-normalized MAR of indicated cell types in late G2 and M-phase. Note that L1210 cells obtained from ECACC did not display an increase in MAR in early mitosis. All other L1210 experiments were done with cells obtained from ATCC. M/A denotes the metaphase-to-anaphase transition, G2/M denotes the approximate mitotic entry, both of which are indicated with dashed vertical lines. Solid dark blue lines indicate the mean and light blue areas represent ± SD. n refers to number of individual cells analyzed. (**c**) Reproducibility of L1210 cell mitotic growth dynamics between different SMR devices and over multiple years. Note that multiple batches of L1210 cells were analyzed over the duration of this study and similar mass accumulation behavior was always observed. n refers to number of individual cells analyzed.

We next examined MAR in other cell types. Although increased MAR during early mitosis was only observed in some cell types, MAR remained high after mitotic entry (during approximate prophase) for all the cell types studied (Figure 2e; Figure 2—figure supplement 2b). All cell types displayed rapid reduction of MAR during metaphase (possibly starting in late prometaphase), near zero MAR during anaphase and a recovery of MAR during late cytokinesis. The temporary stop of cell growth in anaphase was consistently short (<15 min) and coincided with the physical separation of the daughter cells (Figure 2c–e). Notably, some cells displayed a negative MAR, indicating a small loss of cell mass during late metaphase and/or early anaphase (Figure 2e; Figure 2—figure supplement 2b). Together, these results indicate that the mitotic growth behavior is conserved across various animal cell types in suspension, suggesting a role for these specific growth dynamics during mitosis.

### Mitotic protein synthesis rates are consistent with mitotic MAR dynamics

**Figure 3.**
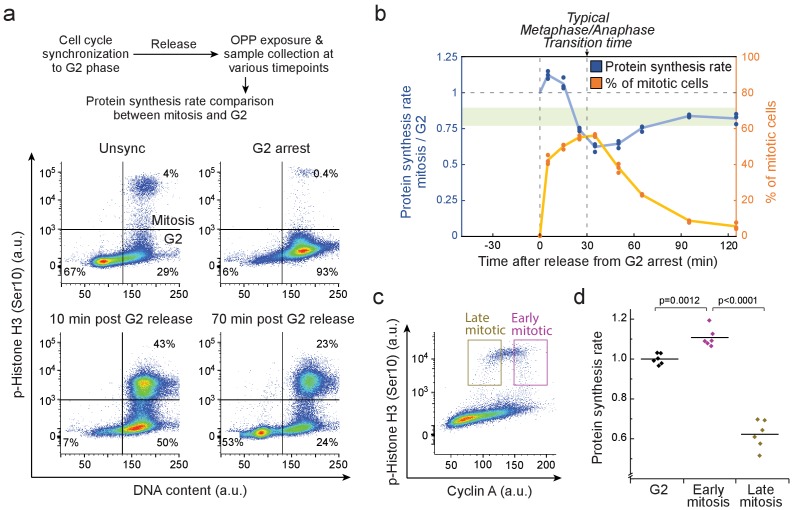
Mitotic protein synthesis dynamics are consistent with mass accumulation dynamics. (**a**) *Top,* schematic of the protocol for quantifying mitotic protein synthesis rates using O-propargyl-puromycin (OPP). G2 synchronization was achieved by double thymidine block followed by RO-3306 mediated G2 arrest. *Bottom,* representative FACS scatter plots indicating L2110 cell cycle synchrony (n = 3 independent experiments). Phospho-Histone H3 (Ser10) was used as a mitotic marker. (**b**) Ratio of protein synthesis rate (blue) between mitotic and G2 L1210 cells after release from G2 arrest. Light green area displays the typical protein synthesis ratio between mitotic and G2 cells in the absence of cell cycle synchronization. The relative portion of mitotic cells is shown in orange. Each data point represents an individual replicate. (n = 3 separate cultures for each timepoint). Time of G2 release and the typical time to reach metaphase-to-anaphase transition are indicated with dashed vertical lines. (**c**) Representative FACS scatter plot indicating the separation of early (prophase) and late (metaphase to telophase) mitotic L1210 cells using Cyclin A antibody staining. (**d**) Protein synthesis rate of G2, early mitotic and late mitotic L1210 cells. (n = 6 separate cultures). Early and late mitotic cells were separated as shown in (**g**). p-Values obtained using ANOVA followed by Tukey’s posthoc test.

Proteins constitute approximately 70% of cellular dry mass (Palm and Thompson, 2017), making it likely that the measured MAR dynamics reflect protein synthesis rates. Using L1210 cells as a model, we quantified the dynamics of mitotic protein synthesis using O-propargyl-puromycin (OPP)-based single-cell protein synthesis assays (Liu et al., 2012) together with a mitotic marker (phospho-Histone H3 (Ser10)) (Figure 3a; Figure 3—figure supplement 1a,b; Materials and methods). For unsynchronized cells, the average mitotic protein synthesis rates were 85 ± 6% (mean ± SD, n = 6) of the rate for G2 cells. We then synchronized cells using double thymidine block followed by CDK1 inhibitor (RO-3306)-mediated G2 arrest and a release, which was followed by 10 min OPP labelling at various timepoints. We normalized mitotic protein synthesis rates to G2 protein synthesis rates to avoid cell synchronization induced biases (Figure 3a). Immediately after the release from G2 arrest, when mitotic cells were in prophase, protein synthesis rates were higher in mitotic cells than in G2 cells (Figure 3b). Approximately 30 min later, when most mitotic cells proceeded to cytokinesis, the protein synthesis rates were reduced to below the normal G2 levels.

**Figure 3—figure supplement 1.**
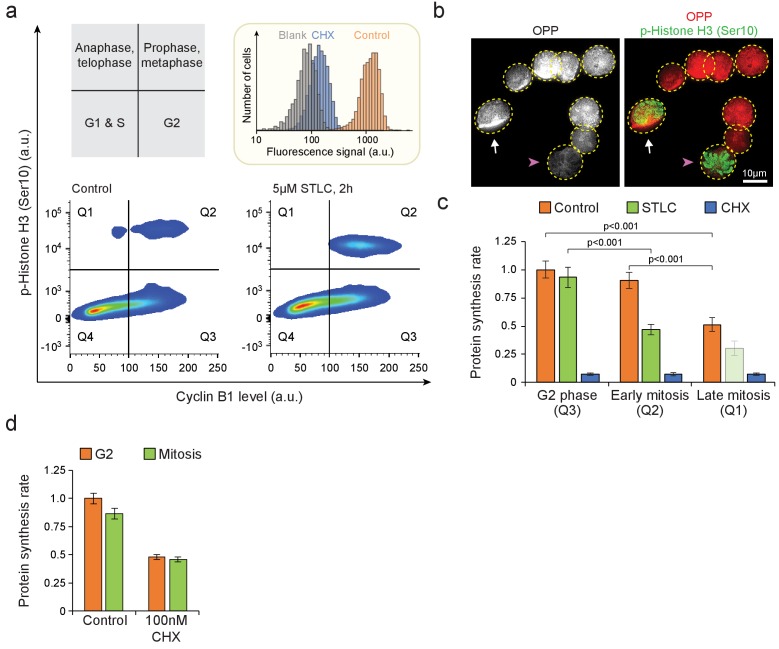
Single-cell protein synthesis assays in mitotic cells. (**a**) *Top left,* schematic indicating the approximate cell cycle stages separated by Cyclin B1 and phospho-Histone H3 (Ser10) antibody labeling. *Bottom,* representative FACS scatter plots indicating the separation of early (prophase to metaphase) and late (anaphase to telophase) mitotic L1210 cells. Arresting cells to metaphase using STLC increases the number of cells in early mitosis but decreases the number of cells in late mitosis, validating the cell cycle separation. *Top right,* a histogram indicating O-propargyl-puromycin (OPP) protein synthesis assay specificity, by comparing control (orange) and 100 µM cycloheximide (CHX) (blue) treated samples. (n = 6 separate cultures for control, three separate cultures for Blank, CHX and STLC samples). (**b**) Representative image of control L1210 cells labeled with OPP and phospho-Histone H3 (Ser10) antibody (n = 12 fields of view). An early mitotic cell (white arrow), with phospho-Histone H3 (Ser10) labeling but not fully compacted chromatin, displays OPP incorporation comparable to OPP incorporation in non-mitotic cells (cells without arrows). A metaphase cell (pink arrowhead), with phospho-Histone H3 (Ser10) labelling and compacted chromatin, displays low OPP incorporation. Dashed yellow lines indicate cell outlines. (**c**) Protein synthesis rates of G2, early mitotic and late mitotic L1210 cells. Early and late mitotic cells were separated as shown in (**a**). Chemical treatment with 5 µM STLC lasted 2 hr and with 100 µM CHX lasted 30 min. Data in late mitosis after STLC treatment is indicated with light green bar, as this population had too few cells for a reliable analysis. (n = 3–6 separate cultures). (**d**) Protein synthesis rates of G2 and mitotic L1210 cells after 4 hr treatment with 100 nM CHX. (n = 4 separate cultures). In (**c**) and (**d**), data depicts mean ± SD. p-Values obtained using ANOVA followed by Tukey’s posthoc test.

To validate that the observed protein synthesis dynamics are not an artefact of cell synchronization, we utilized a previously developed approach where Cyclin A, which is degraded during prometaphase (den Elzen and Pines, 2001), is used to separate early (prophase) and late (metaphase to telophase) mitotic cells (Ly et al., 2017) (Figure 3c). In the absence of cell cycle synchronization, OPP-based protein synthesis assay indicated that early mitotic cells have higher protein synthesis rates than G2 cells, whereas in late mitotic cells, protein synthesis is reduced (Figure 3d). We also separated G2, early mitosis and late mitosis based on cyclin B1, which is degraded at metaphase-to-anaphase checkpoint (Figure 3—figure supplement 1a). This approach also revealed that protein synthesis rates remain high in early mitosis but not in late mitosis (Figure 3—figure supplement 1c). Although protein synthesis assays do not have temporal resolution required for separation of all mitotic stages, the protein synthesis dynamics we observe correspond to those observed with MAR measurements. In conclusion, L1210 cells display increased growth in early mitosis and radically reduced growth in metaphase and anaphase.

### Mitotic arrests, including antimitotic chemotherapies, inhibit cell growth

**Figure 4.**
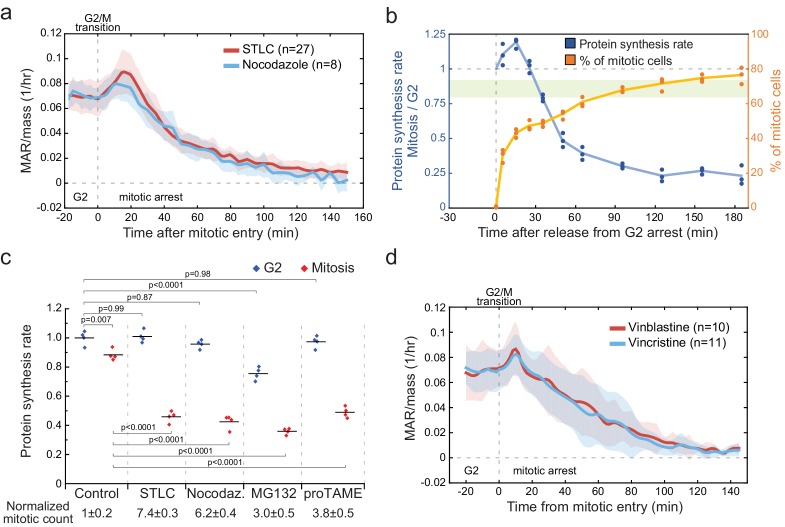
Mitotic arrests result in growth inhibition independently of the mechanism of arrest. (**a**) Mass-normalized MAR of 5 µM STLC or 1 mg/ml Nocodazole treated L1210 cells in late G2 and mitosis. Dashed vertical line indicates the approximate mitotic entry. Solid dark lines indicate the mean and light areas represent ± SD. n refers to number of individual cells analyzed. Drug treatments started 1–4 hr prior to mitotic entry and were maintained through the experiment. (**b**) Ratio of protein synthesis rate (blue) between mitotic and G2 L1210 cells after release from G2 arrest in to 5-µM STLC-mediated mitotic arrest. Light green area displays the typical protein synthesis ratio between mitotic and G2 cells in the absence of cell cycle synchronization. The relative portion of mitotic cells is shown in orange. Each data point represents an individual replicate (n = 3 separate cultures for each timepoint). Cells were synchronized to G2 as in (Figure 3a). (**c**) Protein synthesis rates of G2 (blue) and mitotic (red) L1210 cells after 4 hr treatment with indicated mitotic inhibitors. The proportion of mitotic cells relative to control is indicated below (mean ± SD). (n = 4 separate cultures). p-Values obtained using ANOVA followed by Tukey’s posthoc test. (**d**) Mass-normalized MAR of 10 nM Vinblastine or 10 nM Vincristine-treated L1210 cells in late G2 and mitosis. Dashed vertical line indicates the approximate mitotic entry. Solid dark lines indicate the mean and light areas represent ± SD. n refers to number of individual cells analyzed. Drug treatments started 1–4 hr prior to mitotic entry and were maintained through the experiment.

Our results show that growth is inhibited from metaphase (or late prometaphase) to the end of anaphase (Figure 2a). As many studies examine mitotic growth by arresting cells to mitosis, and this has been suggested to reduce cell growth (Coldwell et al., 2013; Sivan et al., 2011; Stonyte et al., 2018), we measured the effect of chemically induced mitotic arrest on cell growth. First, we monitored the MAR of L1210 cells treated with kinesin inhibitor S-trityl-l-cysteine (STLC), which arrests cells in prometaphase state. These cells displayed a growth burst in early mitosis, similarly to untreated cells, after which MAR approached zero over the course of 2–3 hr (Figure 4a). We also repeated our mitotic protein synthesis assay (Figure 3a) in the presence of STLC. Similarly to MAR, protein synthesis rates increased after mitotic entry, but then gradually decreased as cells were arrested in mitosis (Figure 4b).

To separate drug-specific effects on growth from those that reflect mitotic arrest, we tested three additional chemical approaches for arresting cells in mitosis. These were microtubule inhibitor nocodazole, proteasome inhibitor MG-132 and Anaphase-Promoting Complex inhibitor proTAME (Zeng et al., 2010). All these chemicals resulted in similar reduction in the overall mitotic protein synthesis (Figure 4c). None of these chemicals caused protein synthesis to be significantly reduced in G2, except for MG-132. In addition, nocodazole treatment resulted in identical MAR behavior as STLC (Figure 4a).

Mitotic arrest is the mechanism of action for many chemotherapy drugs. We examined how clinically relevant concentrations of the chemotherapy drugs Vinblastine and Vincristine (Florian and Mitchison, 2016) affect cell MAR. Neither of the drugs affected cell growth in G2, but as cells were arrested in mitosis, their growth rate reduced to zero (Figure 4d). Thus, mitotic arrests, including antimitotic chemotherapies, stop cell growth independently of the arresting mechanism.

### Cells in metaphase and anaphase display mitotic stage specific inhibition of mass accumulation

Next, we studied if metaphase and anaphase, where MAR was near zero, have a growth reducing mechanism(s) that is not active earlier in mitosis. We first considered the role of mitotic deswelling. During mitosis cells round up and increase their volume by approximately 10–20%, before shrinking (deswelling) back to their original volume during anaphase (Son et al., 2015a; Zlotek-Zlotkiewicz et al., 2015). While mitotic rounding has minimal influence on the cell types used in this study, as the suspension cells display a spherical morphology throughout the cell cycle, inhibition of mitotic cell swelling removed the MAR increase seen in early mitosis but did not affect MAR in metaphase and anaphase (Figure 5—figure supplement 1a–c). Furthermore, inhibition of mitotic swelling did not influence early mitotic protein synthesis rates (Figure 5—figure supplement 1d). Thus, while mitotic swelling influences the MAR observed in early mitosis, possibly by increasing cell mass due to the uptake of ions, this does not explain why cells suddenly stop growing around metaphase-to-anaphase transition.

**Figure 5.**
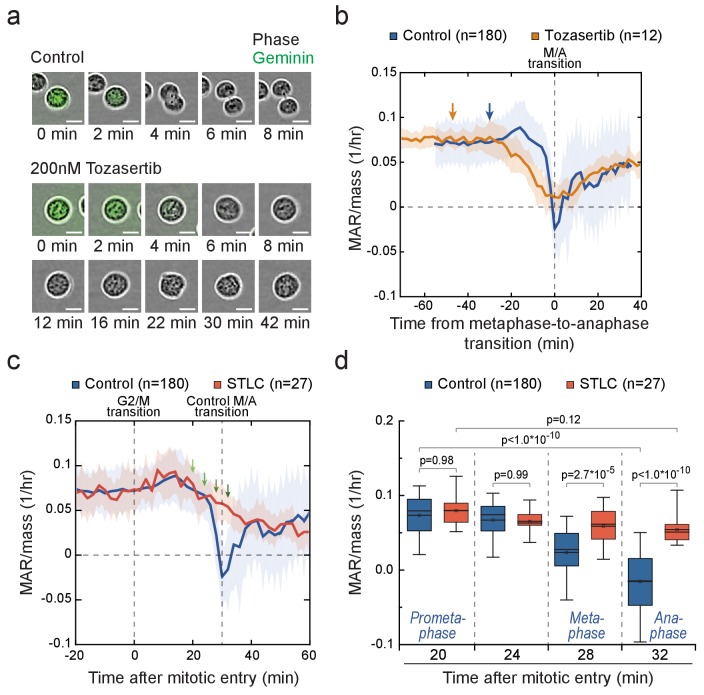
Cells in metaphase and anaphase display stage-specific mass accumulation regulation independently of cell elongation. (**a**) Representative L1210 cell phase contrast (grey) and mAG-hGeminin cell cycle reporter (green) images in control (n = 9 cells) and 200 nM Tozasertib (n = 6 cells) treated cells. The degradation of mAG-hGeminin indicates metaphase-to-anaphase transition. No cytokinesis takes place under Tozasertib treatment. (**b**) Mass-normalized MAR of control (blue) and 200 nM Tozasertib (orange) treated L1210 cells. Note that Tozasertib prolonged early mitosis, but most cells still displayed increased MAR after G2/M transition (see Figure 5—figure supplement 2a). Dashed vertical line indicates the metaphase-to-anaphase transition. Solid dark lines indicate the mean and light areas represent ± SD. n refers to number of individual cells analyzed. Arrows reflect typical time of G2/M transition for each sample. Drug treatment started 1–4 hr prior to mitotic entry and was maintained through the experiment. (**c, d**) Mass-normalized MAR of control (blue) and 5 µM STLC (red) treated L1210 cells (**f**). Dashed vertical lines indicate the approximate mitotic entry (for both samples) and metaphase-to-anaphase transition (only applies to control). Solid dark lines indicate the mean and light areas represent ± SD. Arrows indicate time points from which data was extracted to generate the boxplot in (**g**). n refers to number of individual cells analyzed. p-Values were obtained using ANOVA followed by Tukey’s posthoc test. In all boxplots, line: median, box: interquartile range, whiskers: 5–95%. See Materials and methods for details on MAR analysis resolution for this figure.

Next, we examined if cytokinetic cell elongation is required for the near zero (or even negative) MAR around metaphase-to-anaphase transition. We treated L1210 cells with Tozasertib, an Aurora kinase inhibitor, which blocks cytokinesis but not mitosis as evident from the loss of Geminin (Figure 5a). Tozasertib-treated cells displayed low MAR around metaphase-to-anaphase transition, although MAR remained higher than in control cells (Figure 5b; Figure 5—figure supplement 2a). We then treated cells with blebbistatin, a myosin motor inhibitor which also blocks cytokinesis (Atilla-Gokcumen et al., 2010). Blebbistatin treatment resulted in MAR dynamics comparable to control cells (Figure 5—figure supplement 2b). In addition, both Tozasertib and blebbistatin prolonged the duration of early mitosis. Together, these data indicate that the physical separation of daughter cells does not explain the observed MAR dynamics.

**Figure 5—figure supplement 1.**
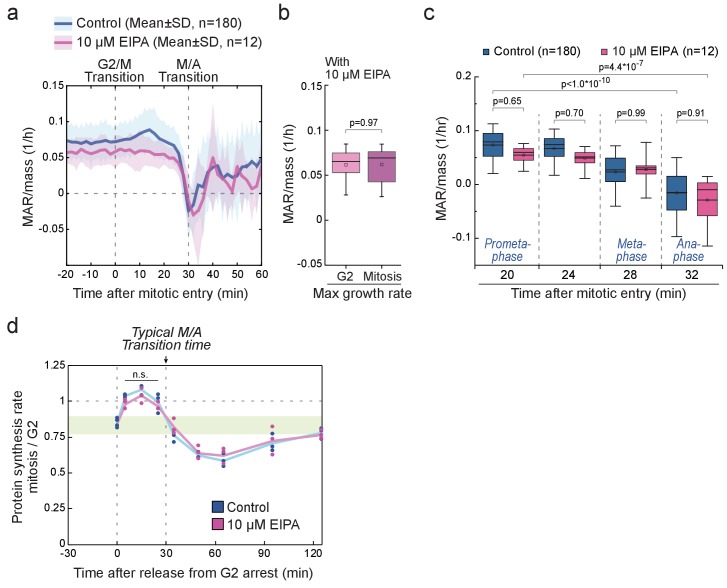
Mitotic cell swelling affects MAR, but not protein synthesis in early mitosis. (**a**) Mass-normalized MAR of control (blue) and 10 µM EIPA (pink) treated L1210 cells. Dashed vertical lines indicate the approximate mitotic entry and metaphase-to-anaphase transition. Solid dark lines indicate the mean and light areas represent ± SD. n refers to number of individual cells analyzed. Drug treatment started 1–4 hr prior to mitotic entry and was maintained through the experiment. (**b**) Quantification of L1210 cell maximal growth rate observed in late G2 and in mitosis when treated with 10 µM EIPA (n = 12 cells, each an independent experiment). p-Values obtained using two-tailed Welch’s t-test. (**c**) Quantifications of data displayed in panel a. Mass-normalized MAR of control (blue) and EIPA (pink)-treated L1210 cells were analyzed as a function of time after mitotic entry. n refers to number of individual cells analyzed. p-Values were obtained using ANOVA followed by Tukey’s posthoc test. (**d**) Ratio of protein synthesis rate between mitotic and G2 L1210 cells after release from G2 arrest in to control (blue) or 10 µM EIPA (pink). Light green area displays the typical protein synthesis ratio between mitotic and G2 cells in the absence of cell cycle synchronization. Each data point represents an individual replicate (n = 3 separate cultures for each timepoint). Cells were synchronized to G2 as in (Figure 3a). In all boxplots, line: median, box: interquartile range, whiskers: 5–95%. See Materials and methods for details on MAR analysis resolution for this figure.

**Figure 5—figure supplement 2.**
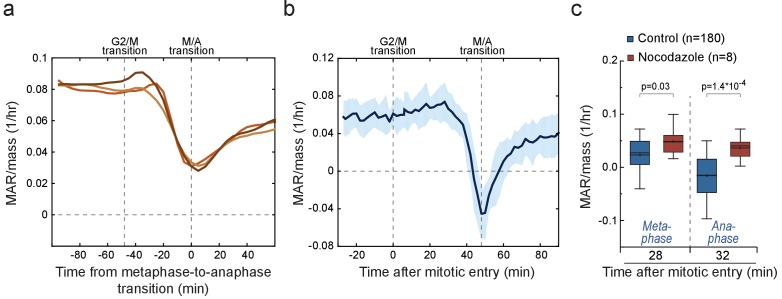
The low MAR in metaphase and anaphase are not explained by cell elongation or time spent in mitosis. (**a**) Examples of individual 200 nM Tozasertib treated L1210 mass-normalized MAR traces. Note that although MAR remains positive around metaphase-to-anaphase transition, the absence of cytokinesis in these cells does not remove the overall dynamics of MAR (See Figure 2d for control dynamics). Dashed vertical lines indicate the approximate mitotic entry and metaphase-to-anaphase transition. Drug treatment started 1–4 hr prior to mitotic entry and was maintained through the experiment. (**b**) Mass-normalized MAR of 25 µM Blebbistatin-treated L1210 cells. Solid dark blue line indicates the mean and light areas represent ± SD (n = 9 individual cells, each from separate experiment). Dashed vertical lines indicate the approximate mitotic entry and metaphase-to-anaphase transition. Drug treatment started 1–4 hr prior to mitotic entry and was maintained through the experiment. (**c**) MAR/mass of control (blue) and Nocodazole (dark red)-treated L1210 cells were analyzed as a function of time after mitotic entry. n refers to number of individual cells analyzed. p-Values obtained using two-tailed Welch’s t-test. In boxplots, line: median, box: interquartile range, whiskers: 5–95%. See Materials and methods for details on MAR analysis resolution for this figure.

The radical reduction in MAR observed as cells approach metaphase-to-anaphase transition could be explained by two separate mechanisms: First, growth may be reduced as a function of time after mitotic entry, or possibly after an initial delay in growth reduction. This hypothesis is supported by the gradual decrease in growth rates following mitotic arrests (Figure 4), possibly because of the inhibition of transcription as DNA condenses. Second, there may be a separate growth inhibiting mechanism(s) specific to metaphase and anaphase. To separate these two options, we compared the MAR as a function of time from mitotic entry in control cells and cells arrested in prometaphase state using STLC (Figure 5c). 20 min after mitotic entry, when cells are in late prometaphase, both samples displayed similar growth rates (Figure 5d). However, 28 min and 32 min after mitotic entry, when control cells had proceeded to metaphase and anaphase, respectively, but STLC-treated cells remained arrested in prometaphase, the control cells displayed lower MAR. Similar results were obtained when prometaphase arrest was achieved using Nocodazole (Figure 5—figure supplement 2c). In conclusion, the mitotic morphological changes (Figure 5a,b; Figure 5—figure supplement 1; Figure 5—figure supplement 2a,b) and the time that cells have spent in mitosis cannot fully explain the observed MAR in metaphase and anaphase. Thus, additional MAR reducing mechanism(s) must exist around metaphase-to-anaphase transition.

### Mitotic growth does not require mTOR activity

**Figure 6.**
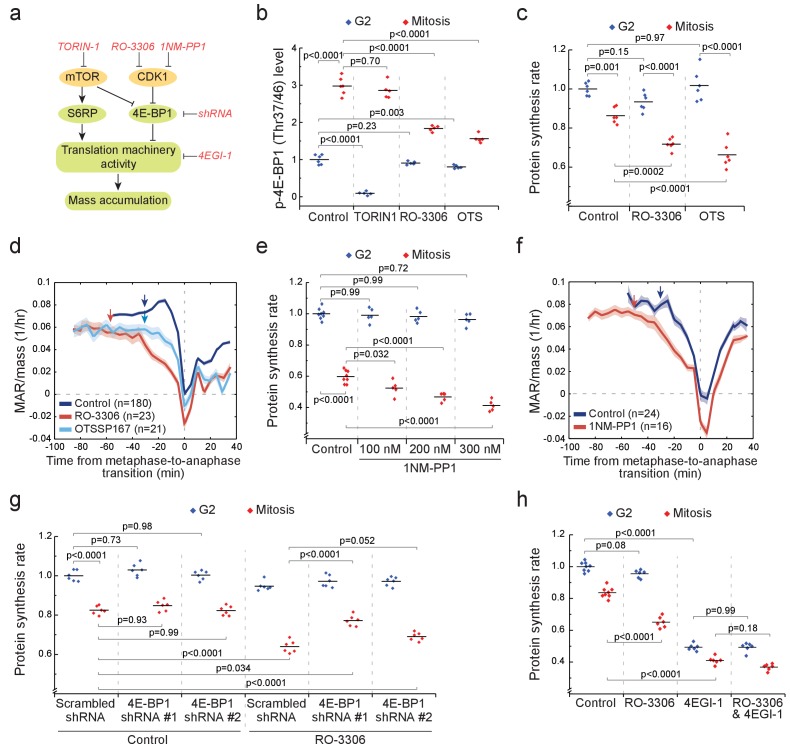
CDK1 drives mitotic growth through 4E-BP1 and cap-dependent protein synthesis. (**a**) Schematic of growth regulation pathways. Chemical and genetic inhibitors (red), kinases (yellow) and the measured downstream consequences (green) are shown. 1NM-PP1-mediated inhibition of CDK1 is dependent on kinase mutation. (**b**) L1210 cell levels of phosphorylated 4E-BP1 (Thr37/46) in G2 (blue) and mitosis (red) after 2 hr treatment with 250 nM TORIN-1, 1 µM RO-3306 or 50 nM OTSSP167. (n = 5–6 separate cultures). (**c**) Protein synthesis rates of G2 (blue) and mitotic (red) L1210 cells after 2 hr treatment with 1 µM RO-3306 or 50 nM OTSSP167. (n = 6 separate cultures). (**d**) Mass-normalized MAR of control, 1 µM RO-3306 or 30 nM OTSSP167-treated L1210 cells. Solid dark lines indicate the mean and light areas represent ± SEM. Arrows reflect typical time of G2/M transition for each sample. n refers to number of individual cells analyzed. Drug treatments started 1–4 hr prior to mitotic entry and were maintained through the experiment. (**e**) DT40 CDK1as cell protein synthesis rates in G2 (blue) and mitosis (red) after 5 hr treatment with 1NM-PP1. (n = 5–8 separate cultures). (**f**) Mass-normalized MAR of control or 200 nM 1NM-PP1-treated DT40 CDK1as cells. Solid dark lines indicate the mean and light areas represent ± SEM. Arrows reflect typical time of G2/M transition for each sample. n refers to number of individual cells analyzed. Drug treatments started 1–4 hr prior to mitotic entry and were maintained through the experiment. (**g**) Protein synthesis rates of G2 (blue) and mitotic (red) L1210 cells expressing scrambled or 4E-BP1 targeting shRNAs. The cells were treated for 2 hr with 1 µM RO-3306 before sample preparation. (n = 6 separate cultures). (**h**) Protein synthesis rates of G2 (blue) and mitotic (red) L1210 cells after 2 hr treatment with 1 µM RO-3306, 5 hr treatment with 50 µM 4EGI-1 or combined treatment with RO-3306 and 4EGI-1. (n = 6–8 separate cultures). All p-Values were obtained using ANOVA followed by Tukey’s posthoc test.

We then investigated what signaling promotes growth and protein synthesis in mitosis (Figure 6a). We measured the levels of phosphorylated 4E-BP1 (Thr37/46) and S6RP (Ser235/236) at the single-cell level in mitotic and G2 L1210 cells. Mechanistic Target Of Rapamycin (mTOR) regulates both of these proteins, which in turn control translation (Fingar et al., 2004). Importantly, 4E-BP1 is a negative regulator of translation and is inactivated by phosphorylation on several sites, including Thr37/46 (reviewed by Qin et al., 2016). Phosphorylated 4E-BP1 levels were approximately threefold higher in mitosis than in G2 (Figure 6b; Figure 6—figure supplement 1), whereas phosphorylated S6RP displayed only a minor increase in mitosis (Figure 6—figure supplement 2a). The mitotic phosphorylation of 4E-BP1 was validated with an independent antibody using microscopy and the mitotic increase was not observed when using antibody isotype controls or pretreating the sample using Lambda protein phosphatase (Figure 6—figure supplement 1). The mitotic phosphorylation of 4E-BP1 is also consistent with previous reports (Shuda et al., 2015) and the observation that the translational targets of mTOR are actively translated during mitosis (Park et al., 2016).

**Figure 6—figure supplement 1.**
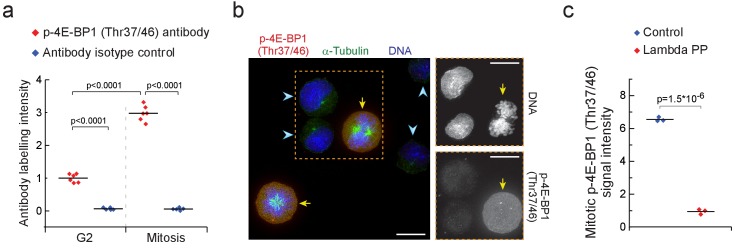
4E-BP1 is phosphorylated in mitosis. (**a**) L1210 cell levels of phosphorylated 4E-BP1 (Thr37/46) in mitosis and G2. Antibody isotype control is shown as a specificity control. (n = 5–6 separate cultures). p-Values obtained using ANOVA followed by Tukey’s posthoc test. (**b**) Representative image of L1210 cells in interphase (light blue arrow heads) and in early mitosis (yellow arrows) with DNA, α-Tubulin and p-4E-BP1 (Thr37/46) labeling (n = 15 fields of view). Mitotic cells were identified based on DNA condensation and α-Tubulin morphology. DNA and p-4E-BP1 (Thr37/46) labeling within the orange box are shown separately on the right. Scale bars denote 10 µm. (**c**) L1210 cell levels of phosphorylated 4E-BP1 (Thr37/46) in mitotic cells treated with or without Lambda protein phosphatase. (n = 3 separate cultures). p-Values obtained using two-tailed Welch’s t-test. See Materials and methods for experimental details.

**Figure 6—figure supplement 2.**
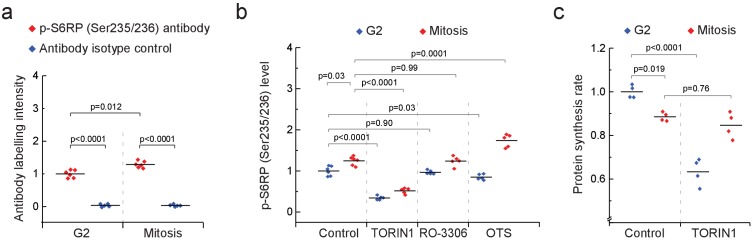
mTOR is active in mitosis but is not required for mitotic protein synthesis. (**a**) L1210 cell levels of phosphorylated S6RP (Ser235/236) in mitosis and G2. Antibody isotype control is shown as a specificity control. (n = 5–6 separate cultures). (**b**) L1210 cell levels of phosphorylated S6RP (Ser235/236) in G2 (blue) and mitosis (red) after 2 hr treatment with 250 nM TORIN-1, 1 µM RO-3306 or 50 nM OTSSP167. (n = 5–6 separate cultures). (**c**) Protein synthesis rates of G2 (blue) and mitotic (red) L1210 cells after 2 hr treatment with 250 nM TORIN-1. (n = 4 separate cultures). All p-values obtained using ANOVA followed by Tukey’s posthoc test.

**Figure 6—figure supplement 3.**
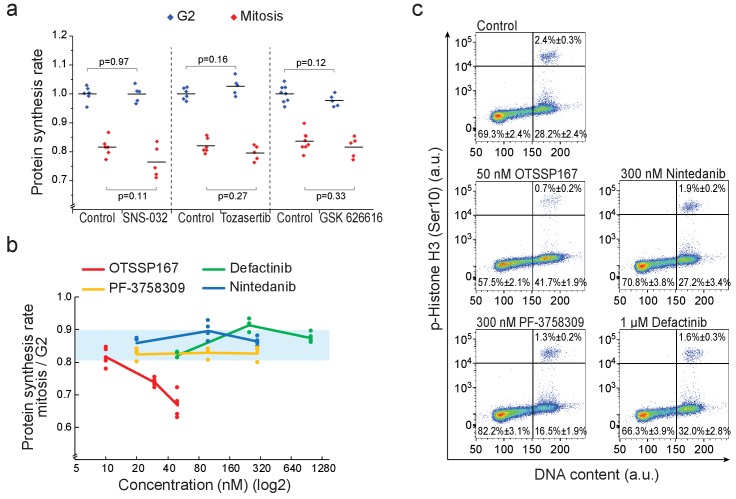
Kinase inhibitors which have MELK kinase as an off-target do not reduce mitotic protein synthesis. (**a**) Protein synthesis rates of G2 (blue) and mitotic (red) L1210 cells after 3 hr treatment with 1 µM SNS-032 (CDK2 inhibitor) or 50 nM Tozasertib (Aurora kinase inhibitor), or 5 hr treatment with 1 µM GSK 626616 (DYRK kinase inhibitor). (n = 5–8 separate cultures). p-Values obtained using two-tailed Welch’s t-test. (**b**) Ratio of protein synthesis rates between mitotic and G2 L1210 cells after 3 hr treatment with OTSSP167, Defactinib (also known as PF-04554878), PF-3758309 (also known as PF-309) or Nintedanib (also known as BIBF 1120), all of which inhibit MELK kinase with sub-micromolar affinity (Klaeger et al., 2017). Light blue area indicates the typical range observed in control cells. (n = 4 separate cultures). Note that OTSSP167 is a nonspecific multikinase inhibitor (Klaeger et al., 2017), making it likely that MELK kinase does not contribute to the drug-induced mitotic growth reduction. (**c**) Representative FACS scatter plots indicating the cell cycle distribution after 6 hr treatment with indicated chemicals. The relative portion of cells in G1/S, G2 and mitosis are indicated (mean ± SD). (n = 4–6 separate cultures). Note that OTSSP167 reduced mitotic entry, a phenotype typical for CDK1 inhibition, while the alternative MELK inhibitors did not display similar cell cycle profile. CDK1 is one of the off-targets of OTSSP167 (Klaeger et al., 2017).

**Figure 6—figure supplement 4.**
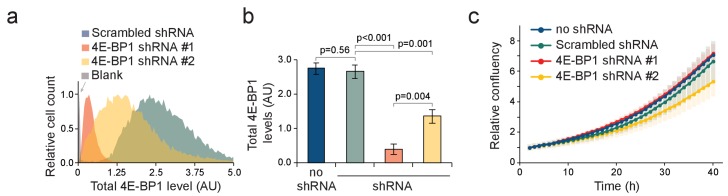
shRNA-mediated knockdown of 4E-BP1 in L1210 cells. (**a**) Histogram of 4E-BP1 protein levels analyzed by immunostaining and flow cytometry in L1210 cells stably expressing the indicated shRNAs. (**b**) Quantifications of data in panel (**a**). n = 3 separate cultures. Note that the difference in 4E-BP1 knockdown efficiency between the two 4E-BP1 targeting shRNAs is likely to explain the differences observed between these two cell lines in Figure 6e. (**c**) Proliferation rate of control and 4E-BP1 knockdown cells. Note that the lack of strong proliferation phenotypes likely reflects the optimal growth conditions where 4E-BP1 is maintained phosphorylated and inactivated.

To examine the role of mTOR, we treated cells for 2 hr with 250 nM mTOR inhibitor TORIN-1. In G2 cells, the levels of phosphorylated 4E-BP1 (Thr37/46) and S6RP (Ser235/236) were near zero (Figure 6b; Figure 6—figure supplement 2b). In mitosis, phosphorylation of S6RP was reduced by TORIN-1 (Figure 6—figure supplement 2b), indicating that mTOR remains active in mitosis. However, TORIN-1 did not change the levels of phosphorylated 4E-BP1 in mitosis (Figure 6b), suggesting that an mTOR-independent mechanism activates 4E-BP1 in mitosis. Next, we measured mitotic protein synthesis rates in the presence of TORIN-1. Although G2 protein synthesis rates were reduced, mitotic protein synthesis rates were not affected by TORIN-1 (Figure 6—figure supplement 2c). Thus, mTOR is not a major contributor to mitotic growth.

### CDK1 drives phosphorylation of 4E-BP1, protein synthesis and mass accumulation in mitosis

We examined how the levels of phosphorylated 4E-BP1 (Thr37/46) and S6RP (Ser235/236) change when CDK1 is inhibited with 1 µM RO-3306 (Vassilev et al., 2006). At this RO-3306 concentration, L1210 cells can still progress through mitosis, as CDK1 is only partially inhibited (Son et al., 2015a). Only in mitosis did RO-3306 reduce phosphorylated 4E-BP1 but not phosphorylated S6RP (Figure 6b; Figure 6—figure supplement 2b). Although the role of CDK1 in controlling 4E-BP1 phosphorylation has been reported before (Heesom et al., 2001; Shuda et al., 2015), the consequences of this on protein synthesis and cell growth remain unknown. We observed that 1 µM RO-3306 treatment also reduced mitotic protein synthesis rates without affecting G2 protein synthesis rates (Figure 6c). In addition, RO-3306 treatment prolonged early mitosis and resulted in a clear reduction of MAR in mitosis but not in G2 (Figure 6d).

To further validate the role of CDK1 in promoting mitotic growth, we utilized chemical genetics in the DT40 CDK1as cell line. In these cells, the wild-type CDK1 has been replaced with *Xenopus laevis* CDK1 that has a F80G mutation (Gibcus et al., 2018), which sensitizes CDK1 to inhibition by the ATP analog 1NM-PP1 (Hochegger et al., 2007). We observed that protein synthesis was reduced by 1NM-PP1 in a dose-dependent manner in mitosis but not in G2 (Figure 6e). Consistently, 1NM-PP1 also reduced MAR in mitosis (Figure 6f).

We also investigated the role of other mitotic kinases in promoting protein synthesis and cell growth. Inhibitors for CDK2, Aurora kinases and DYRK kinases did not affect mitotic protein synthesis (Figure 6—figure supplement 3a). OTSSP167, a drug designated as a MELK inhibitor (Chung et al., 2012), reduced phosphorylation of 4E-BP1, protein synthesis and mass accumulation rates in mitosis (Figure 6b–d). Consistently, MELK has been suggested to promote translation in mitosis (Wang et al., 2016). However, alternative MELK inhibitors (Klaeger et al., 2017) did not display mitosis-specific inhibition of protein synthesis (Figure 6—figure supplement 3b,c), suggesting that the mitotic growth effects observed in OTSSP167-treated cells were not mediated by MELK but by OTSSP167 off-targets.

We then moved to validate that 4E-BP1 mediates the CDK1 driven protein synthesis. We generated two L1210 cell lines expressing 4E-BP1 targeting shRNAs, which reduced 4E-BP1 levels by approximately 85% (shRNA #1) and by 50% (shRNA #2) (Figure 6—figure supplement 4a,b). The 4E-BP1 knockdowns had little effect on proliferation rate (Figure 6—figure supplement 4c) or G2 cell protein synthesis (Figure 6g), possibly reflecting that 4E-BP1 is kept mostly inactive under our experimental conditions. However, when we reduced mitotic protein synthesis by inhibiting CDK1 with RO-3306, the knockdowns of 4E-BP1 partially rescued the mitotic protein synthesis inhibition (Figure 6g). These data indicate that CDK1 promotes mitotic growth at least partly through 4E-BP1.

4E-BP1-driven cap-dependent protein synthesis is classically considered to be inhibited in mitosis (Bonneau and Sonenberg, 1987; Pyronnet et al., 2001), although recently this view has been challenged (Coldwell et al., 2013; Shuda et al., 2015). We therefore tested if i) cap-dependent translation remains active in L1210 cell mitosis, and if ii) cap-dependent translation is required for CDK1-mediated mitotic growth. To test these, we first inhibited cap-dependent translation using 4EGI-1, an inhibitor of eIF4F complex assembly (Cencic et al., 2011). Both G2 and mitotic protein synthesis rates were reduced by a similar amount, approximately 50%, following 4EGI-1 treatment (Figure 6h). However, treatment with both 4EGI-1 and RO-3306 did not significantly change mitotic protein synthesis rates when compared to treatment with 4EGI-1 alone, suggesting that cap-dependent protein synthesis is involved in CDK1-driven mitotic translation. In conclusion, our results are consistent with a previous report (Shuda et al., 2015) that CDK1 substitutes for mTOR in mitosis to promote phosphorylation of 4E-BP1, to maintain cap-dependent translation and to promote mass accumulation.

### CDK1-driven mitotic protein synthesis supports daughter cell growth

**Figure 7.**
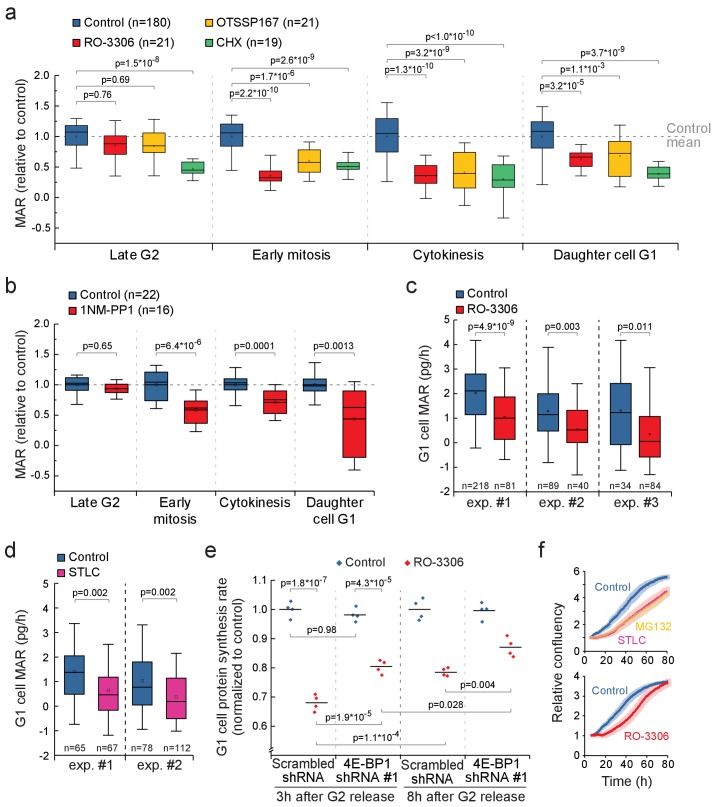
CDK1-driven mitotic protein synthesis supports daughter cell growth. (**a**) MAR during indicated stages of cell cycle in control, 1 µM RO-3306, 30 nM OTSSP167 or 100 nM cycloheximide (CHX) treated L1210 cells. The MAR values were normalized to control mean at each stage. n refers to number of individual cells analyzed. Drug treatments started 1–4 hr prior to mitotic entry and were maintained through the experiment. (**b**) MAR during indicated stages of cell cycle in control or 200 nM 1NM-PP1-treated DT40 CDK1as cells. The MAR values were normalized to control mean at each stage. n refers to number of individual cells analyzed. Drug treatment started 1–4 hr prior to mitotic entry and was maintained through the experiment. (**c** and **d**) MAR of newborn L1210 G1 cells from control and from cells that have undergone mitosis in the presence of 1 µM RO-3306 (**c**) or from cells that have been arrested to mitosis for 4 hr with STLC before releasing to undergo cytokinesis (**d**). Data acquired using serial SMR (see Figure 7—figure supplement 2 for details). n refers to number of individual cells analyzed. (**e**) Protein synthesis rates of G1 L1210 cells expressing a scrambled or 4E-BP1 targeting shRNA after the cells progressed through mitosis in the presence or absence of 1 µM RO-3306. Two timepoints (3 hr and 8 hr) after G2 release are shown. (n = 4 separate cultures). (**f**) Long-term growth, as measured by cell confluency, in L1210 cells that have been arrested to mitosis for 4 hr with STLC or MG132 before releasing to undergo cytokinesis (n = 5 separate cultures, top), or have undergone mitosis in the presence of 1 µM RO-3306 (n = 6 separate cultures, bottom). Dark colors indicate mean and light areas indicate ± SEM. In (**a**) and (**e**), p-values obtained using ANOVA followed by Tukey’s posthoc test. In (**b–d**), p-values obtained using two-tailed Welch’s t-test. In boxplots, line: median, box: interquartile range, whiskers: 5–95%.

Mitotic transcription and translation have been suggested to be geared toward ribosomal proteins and other components that promote growth (Aviner et al., 2017; Palozola et al., 2017). Therefore, inhibiting mitotic protein synthesis could also impact growth in cytokinesis and in daughter cells. We compared the MAR of RO-3306 or OTSSP167-treated L1210 cells to control cells in late G2 (last 30 min before G2/M transition), early mitosis (before metaphase-to-anaphase transition), cytokinesis (after metaphase-to-anaphase transition) and newborn G1 (first 30 min after abscission of daughter cells). In the presence of the mitotic growth inhibitors, G2 MAR was not affected, but MAR remained low in cytokinesis and in newborn G1 (Figure 7a). In contrast, cells treated with 100 nM cycloheximide, a translation inhibitor which at this concentration reduces total protein synthesis by approximately 50% (Figure 3—figure supplement 1d), did not display similar cell cycle specificity in growth inhibition (Figure 7a). In addition, mother cell MAR in early mitosis and daughter cell MAR in early G1 correlated in both control (R^2^ = 0.42) and RO-3306 (R^2^ = 0.33) treated cells (Figure 7—figure supplement 1). 1NM-PP1-mediated inhibition of CDK1 in the DT40 CDK1as cell line also resulted in reduced growth during cytokinesis and in daughter cells (Figure 7b).

**Figure 7—figure supplement 1.**
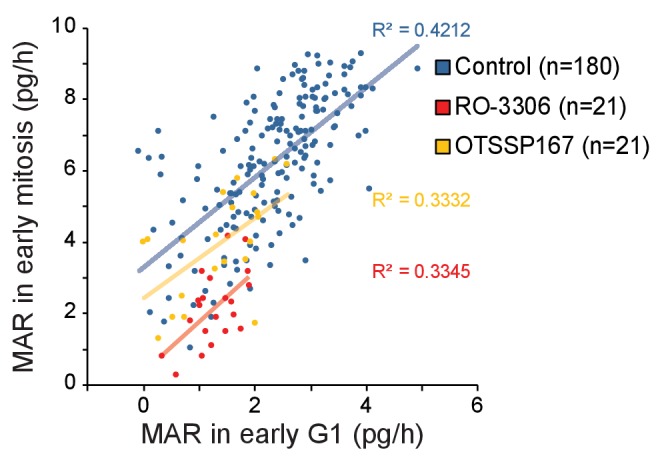
Correlations between mitotic and G1 cell MAR. Correlation plot between mother cell MAR in early mitosis (30 min section prior to M/A transition) and daughter cell MAR in early G1 (first 30 min after abscission) for control, 1 µM RO-3306 and 50 nM OTSSP167 treatments. R-squared values are depicted on right.

**Figure 7—figure supplement 2.**
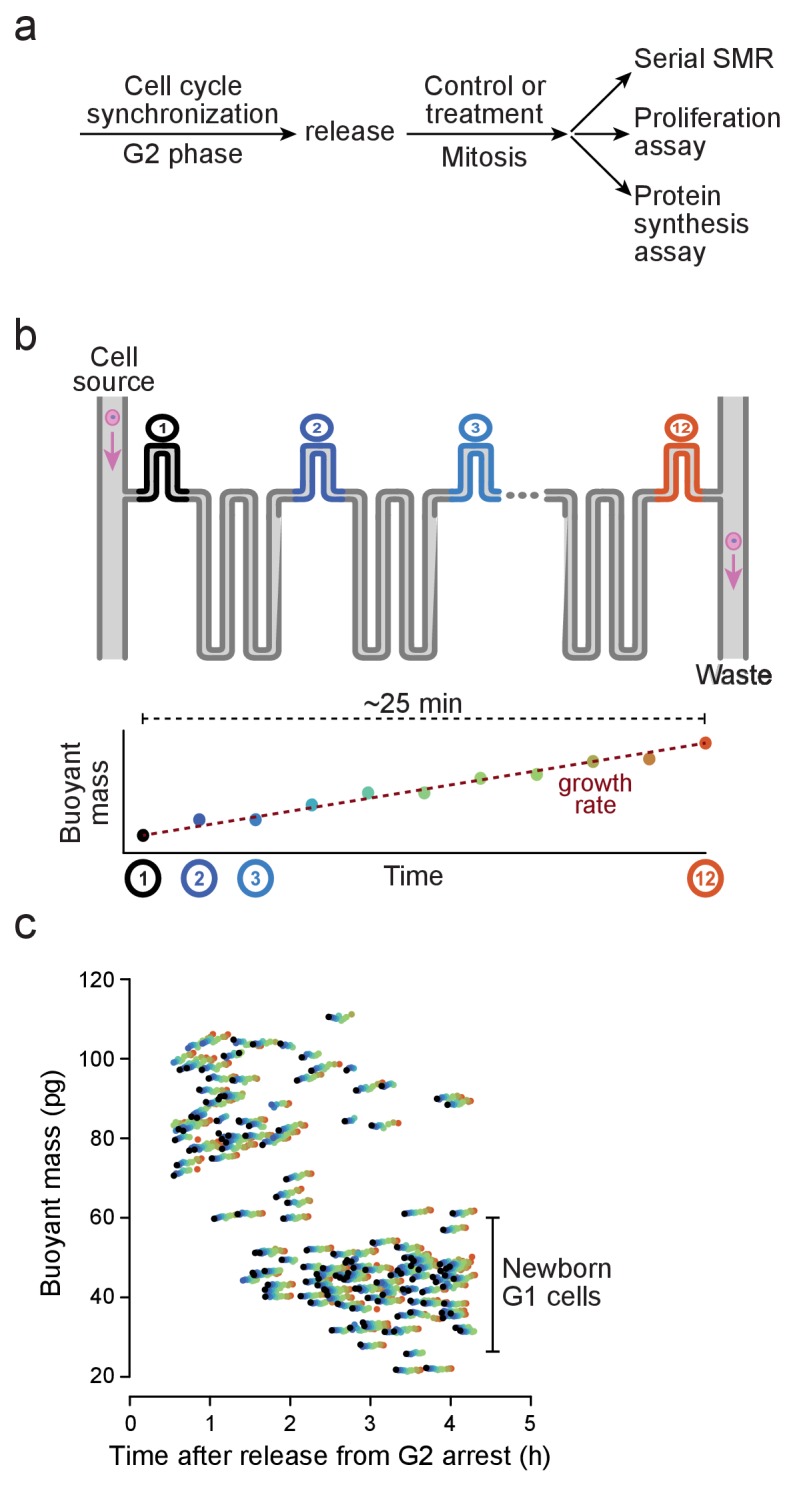
Daughter cell growth rate measurement workflow. (**a**) Workflow for measuring MAR, protein synthesis and proliferation rate of G1 cells that have passed through mitosis under control or drug perturbed conditions. Mitotic RO-3306 treatments were washed away 3 hr after G2 release. (**b**) *Top,* schematic of serial SMR. Cells (pink circles) flow through 12 mass sensing cantilevers separated by delay channels to obtain consecutive mass measurements before being discarded. *Bottom,* the consecutive mass measurements (colored data points) are used to assign a growth (mass accumulation) rate (dashed red line) for each cell. The serial SMR does not allow long-term monitoring of growth but enables much higher MAR measurement throughput than the single cantilever SMR shown in (Figure 1—figure supplement 1a). (**c**) Representative serial SMR data from control L1210 cells (n = 4 independent experiments). G2 synchronized cells were released and the single-cell MAR was monitored by continuously sampling the population. Buoyant mass measured at each cantilever is displayed with different color, as shown at the bottom of (**a**). The G1 cell growth rates can be quantified by analyzing MAR only from the small (25 to 60 pg) cells.

**Figure 7—figure supplement 3.**
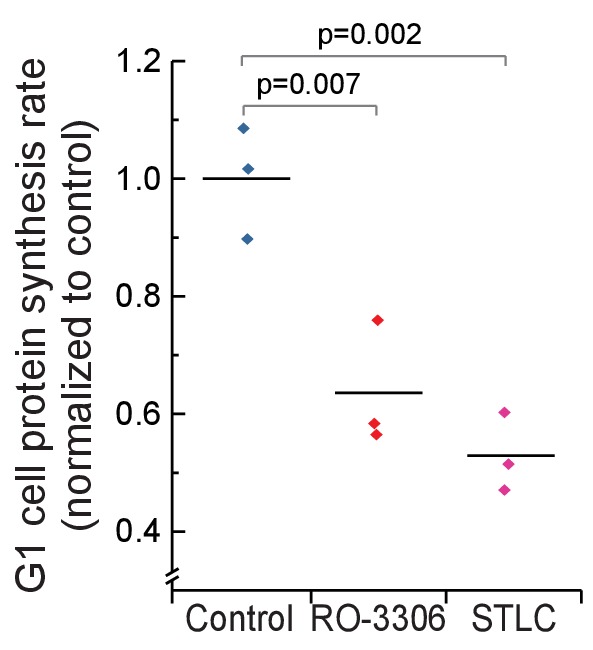
Daughter cells protein synthesis rates in G1 following mitotic growth inhibitions. Protein synthesis rates of G1 L1210 cells from control (normal mitosis) and from cells that have undergone mitosis in the presence of 1 µM RO-3306 or from cells that have been arrested to mitosis for 4 hr with STLC before releasing to undergo cytokinesis. (n = 3 separate cultures).

We speculated that the daughter cell growth inhibition is a consequence of growth reduction in mother cell mitosis. To validate that mitotic growth is needed to promote daughter cell growth, we synchronized L1210 cells to G2 phase, released them back to cell cycle in the presence or absence of mitotic growth inhibitions, and carried out daughter cell growth measurements (see Figure 7—figure supplement 2a for workflow). First, we measured single-cell MAR using a serial SMR, which measures MAR over a 15–30 min window (Calistri et al., 2018; Cermak et al., 2016) (Figure 7—figure supplement 2b). We identified the newborn G1 cells based on their smaller mass and quantified their MAR (Figure 7—figure supplement 2c). In addition, we quantified protein synthesis rates of G1 daughter cells, as identified through DNA staining, and also measured long-term proliferation of the cells. Following mitotic growth inhibitions, either by temporary mitotic arrest (4 hr STLC treatment) or by partial CDK1 inhibition, newborn cells displayed significantly reduced MAR and protein synthesis rates (Figure 7c,d; Figure 7—figure supplement 3). We then repeated the daughter cell protein synthesis assays in 4E-BP1 knockdown cells after the mother cells had progressed through mitosis in the presence or absence of CDK1 inhibition. 4E-BP1 knockdown partly rescued the daughter cell protein synthesis rates that were reduced by CDK1 inhibition (Figure 7e). Thus, 4E-BP1 mediates mitotic protein synthesis of CDK1 (Figure 6), and this may also affect daughter cell protein synthesis. However, we cannot fully exclude the possibility that daughter cell growth is affected by some other effects of partial CDK1 inhibition, such as chromosome missegregation, which could consequently reduce daughter cell growth independently of mitotic growth. The protein synthesis rate and long-term proliferation rate of the daughter cells recovered over time (Figure 7e,f), suggesting that mitotic growth inhibitions do not permanently affect cell viability.

## Discussion

We show that animal cells grow approximately 10% of their total mass during M-phase (Figure 1), indicating that the growth during M-phase is comparable to interphase when the short duration of M-phase is taken into consideration. This contradicts the classical dogma that growth takes place primarily during interphase and not during M-phase. Our single-cell measurements show that mass accumulation behavior during mitosis is dynamic (Figure 2), dependent on mitotic stage (Figure 5), conserved across a variety of animal cell types grown in suspension (Figure 2) and reflected in protein synthesis rates (Figure 3). Importantly, growth is only stopped for a short duration in mitosis, as cells approach the metaphase-to-anaphase transition, and growth recovers in late cytokinesis after anaphase. Most studies on translation and growth signaling during mitosis use cell-population-based experimental approaches that require population enrichment for mitotic cells. This is commonly achieved by mitotic blockades, which result in growth inhibition (Figure 4). Alternatively, mitotic cells can be collected with mitotic shake-off. However, this only enriches for the small subset of mitotic cells that are temporarily not growing (i.e. metaphase and anaphase cells). Thus, many of the controversial reports regarding translational control during mitosis can be explained by experimental approaches.

Conceptually, the tight and conserved coordination between growth rates and mitotic stages suggests that mitotic growth control is important for cell division. It has been thought that prioritization of energy away from the ATP consuming macromolecule synthesis and towards mechanical reorganization of the cell could explain the reduction in mitotic protein synthesis rates (Kronja and Orr-Weaver, 2011; Sivan and Elroy-Stein, 2008; White-Gilbertson et al., 2009). Consistent with this hypothesis, we show that growth is stopped when the physical separation of daughter cells takes place (Figure 2). Yet, there is no direct evidence that cell division would increase the energetic needs of a cell to a point where growth could not persist, and the inhibition of growth may therefore be due to other reasons. For example, the inhibition of growth may protect cells from harmfully excessive cell growth during prolonged mitosis (Miettinen and Björklund, 2016; Miettinen et al., 2017; Neurohr et al., 2019).

It has also been proposed that growth, and especially protein synthesis, are required during mitosis. A complete growth inhibition for the duration of M-phase would result in loss of short-lived proteins needed for mitosis and cytokinesis (White-Gilbertson et al., 2009). Consistently, we observe that growth is not inhibited during prophase or late cytokinesis, suggesting that short-lived proteins, such as survivin (White-Gilbertson et al., 2009), can be produced immediately prior to and after anaphase. Future studies examining cellular energy production and mapping the levels of short-lived proteins in different mitotic stages will help elucidate why cells display such a dynamic growth behavior in mitosis.

Mechanistically, we found that CDK1 promotes mitotic mass accumulation and protein synthesis, and this is at least partly mediated by 4E-BP1 (Figure 6). Our results are consistent with previously reported interactions between CDK1 and the translational machinery (Heesom et al., 2001; Shuda et al., 2015; Smith and Proud, 2008). Importantly, our results do not exclude the existence of other CDK1 dependent or independent mechanisms regulating mitotic growth. Indeed, CDK1 remains active until metaphase-to-anaphase transition (Gavet and Pines, 2010), whereas mass accumulation rates begin to decay in late prometaphase and metaphase, indicating that CDK1 alone cannot fully explain the observed mass accumulation dynamics.

Mitotic growth may be dependent on the time that cells have spent in mitosis, as growth can be limited by the reduced chromatin accessibility and consequently reduced transcription. This is consistent with the gradual growth reduction we observe during mitotic arrests (Figure 4). However, considering the long half-life of most mRNAs (Schwanhäusser et al., 2011), and the observations that mRNA levels do not decline during mitosis (Novais-Cruz et al., 2018; Tanenbaum et al., 2015), limited chromatin accessibility does not explain why protein synthesis would be reduced in the absence of mitotic arrests. Furthermore, our results also show that normal mitotic progression results in more radical reduction in mass accumulation than what is observed in prometaphase arrested cells (Figure 5), indicating that there are also other mechanism(s) that radically reduce mass accumulation around metaphase-to-anaphase transition.

Cell mass accumulation reflects the flux of components, such as nutrients, in and out of the cell. Because we observe that mass accumulation fully stops during anaphase, or even becomes negative in some cell types, the mechanism(s) controlling mass accumulation around metaphase-to-anaphase transition must either block the cells from taking up nutrients or even expel cellular components. Yet, this does not necessarily mean that protein synthesis comes to a complete stop, as cells can maintain translation even in the absence of nutrient uptake by degrading proteins and recycling the components (Son et al., 2015b). In fact, prolonged mitotic arrests resulted in near zero mass accumulation although ~25% of the protein synthesis rate persisted (Figure 4). We therefore hypothesize that around metaphase-to-anaphase transition, where the anaphase-promoting complex drives protein degradation, cells largely stop nutrient uptake but maintain low level of protein synthesis by recycling amino acids.

We also observed that reducing CDK1 activity results in reduced growth in daughter cells (Figure 7). This may be explained by CDK1-driven translation of growth components during mitosis, such as ribosomal proteins, that are required to ‘jump-start’ growth in the newborn cells. Consistently, several studies have suggested that transcription and translation of growth-related genes are prioritized during mitosis (Aviner et al., 2017; Palozola et al., 2017; Park et al., 2016). These results imply that CDK1 does not only coordinate cell division, but also optimizes mitotic translation to promote immediate daughter cell growth. However, it should be noted that the active mitotic translation of growth promoting components remains controversial (Stumpf et al., 2013; Tanenbaum et al., 2015), CDK1 inhibition may reduce daughter cell growth independently of mitotic growth, and the physiological significance of mitotic translational control in vivo remains unexplored.

Finally, our observations that mitotic arrests block cell growth have implications for antimitotic cancer chemotherapy. We observed that the vinca alkaloids Vincristine and Vinblastine, both commonly used to treat lymphomas, stop cell growth when used in concentrations lower than those measured in patient plasma (Florian and Mitchison, 2016). This mitotic growth arrest may contribute to the induction of mitotic catastrophe and the efficacy of antimitotic chemotherapies.

## Materials and methods

**Key resources table:** 

Reagent type (species) or resource	Designation	Source or reference	Identifiers	Additional information
Cell line (*M. musculus*)	L1210	ATCC	Cat#CCL-219; RRID:CVCL_0382	
Cell line (*M. musculus*)	L1210 - FUCCI	Other		Generated in a previous study (Son et al., 2012), Nature Methods), cells originate from ATCC (Cat#CCL-219).
Cell line (*M. musculus*)	L1210 - ECACC	ECACC	Cat#87092804	
Cell line (*M. musculus*)	Fl5.12	Other		Kindly provided by laboratory of Prof. Matthew Vander Heiden from MIT
Cell line (*M. musculus*)	BaF3	RIKEN BioResource Center	Cat#RCB4476	
Cell line (*G. gallus*)	DT40-CDK1as	Other		Kindly provided by laboratory of Prof. Bill Earnshaw from University of Edinburgh
Cell line (*H. sapiens*)	S-HeLa	Other		Kindly provided by laboratory of Kevin Elias from Brigham And Women's Hospital
Transfected construct (*M. musculus*)	Scrambled shRNA	VectorBuilder	Cat#LVS (VB151023-10034)	Refers to a lentiviral construct used to transfect and express the indicated shRNA.
Transfected construct (*M. musculus*)	4E-BP1 shRNA #1	VectorBuilder	Cat#LVS (VB181217-1124dqm)-C	Refers to a lentiviral construct used to transfect and express the indicated shRNA.
Transfected construct (*M. musculus*)	4E-BP1 shRNA #2	VectorBuilder	Cat#LVS (VB181217-1125ypy)-C	Refers to a lentiviral construct used to transfect and express the indicated shRNA.
Biological sample (*H. sapiens*)	Unpurified buffy coat for isolation of T-cells	Research Blood Components	NA	
Antibody	Phospho-Histone H3 (Ser10) (D2C8) XP Rabbit monoclonal Ab (Alexa Fluor 647 Conjugate)	Cell Signaling Technology	Cat#3458; RRID:AB_10694086	Dilution 1/100 in PBS supplemented with 5% BSA
Antibody	Phospho-Histone H3 (Ser10) (D2C8) XP Rabbit monoclonal Ab (Alexa Fluor 488 Conjugate)	Cell Signaling Technology	Cat#3465; RRID:AB_10695860	Dilution 1/100 in PBS supplemented with 5% BSA
Antibody	Cyclin B1 (V152) Mouse monoclonal Ab (Alexa Fluor 488 Conjugate)	Cell Signaling Technology	Cat#4112; RRID:AB_491024	Dilution 1/50 in PBS supplemented with 5% BSA
Antibody	Phospho-4E-BP1 (Thr37/46) (236B4) Rabbit monoclonal Ab (PE Conjugate)	Cell Signaling Technology	Cat#7547; RRID:AB_10949897	Dilution 1/100 in PBS supplemented with 5% BSA
Antibody	Phospho-S6 Ribosomal Protein (Ser235/236) (D57.2.2E) XP Rabbit monoclonal Ab (Alexa Fluor 647 Conjugate)	Cell Signaling Technology	Cat#4851; RRID:AB_10695457	Dilution 1/100 in PBS supplemented with 5% BSA
Antibody	Rabbit (DA1E) monoclonal Ab IgG XP Isotype Control (PE Conjugate)	Cell Signaling Technology	Cat#5742; RRID:AB_10694219	Dilution 1/100 in PBS supplemented with 5% BSA
Antibody	α-Tubulin (11H10) Rabbit monoclonal Ab (Alexa Fluor 488 Conjugate)	Cell Signaling Technology	Cat#5063; RRID:AB_10694858	Dilution 1/200 in PBS supplemented with 5% BSA
Antibody	Cyclin A Mouse monoclonal Ab (H-3) (FITC Conjugate)	Santa Cruz Biotechnology	Cat#sc-271645; RRID:AB_10707658	Dilution 1/25 in PBS supplemented with 5% BSA
Antibody	4E-BP1 (53H11) Rabbit monoclonal Ab (PE Conjugate)	Cell Signaling Technology	Cat#34470	Dilution 1/100 in PBS supplemented with 5% BSA
Antibody	Phospho-4EBP1 (Thr37, Thr46) Rabbit monoclonal Ab (4EB1T37T46-A5)	Thermo Fisher Scientific	Cat#MA5-27999; RRID:AB_2745012	Dilution 1/100 in PBS supplemented with 5% BSA
Antibody	Goat polyclonal anti-Rabbit IgG (H + L) Cross-Adsorbed Secondary Antibody (Alexa Fluor 568 Conjugate)	Thermo Fisher Scientific	Cat#A-11011; RRID:AB_143157	Dilution 1/1000 in PBS supplemented with 5% BSA
Commercial assay or kit	Click-iT Plus OPP Alexa Fluor 594 Protein Synthesis Assay Kit	Thermo Fisher Scientific	Cat#C10457	
Commercial assay or kit	T-cell isolation kit	Miltenyi Biotec	Cat#130-096-495	
Commercial assay or kit	Lambda Protein Phosphatase	New England Biolabs	Cat#P0753S	
Chemical compound, drug	O-propargyl-puromycin (OPP)	Jena Bioscience	Cat#NU-931–5; CAS:1416561-90-4	
Chemical compound, drug	S-trityl-l-cysteine (STLC)	Sigma-Aldrich	Cat#164739; CAS:2799-07-7	
Chemical compound, drug	Nocodazole	Sigma-Aldrich	Cat#M1404; CAS:31430-18-9	
Chemical compound, drug	MG132	Sigma-Aldrich	Cat#474787; CAS:133407-82-6	
Chemical compound, drug	proTAME	Thermo Fisher Scientific	Cat#I44001M; CAS:1362911-19-0	
Chemical compound, drug	Vinblastine	Cayman Chemical	Cat#11762; CAS:143-67-9	
Chemical compound, drug	Vincristine	Cayman Chemical	Cat#11764; CAS:2068-78-2	
Chemical compound, drug	TORIN-1	Tocris Bioscience	Cat#4247; CAS: 1222998-36-8	
Chemical compound, drug	RO-3306	Cayman Chemical	Cat#15149; CAS:872573-93-8	We observed the chemical loses its activity within a month when stored in −20°C. All experiments were done using a stock under 2 weeks old.
Chemical compound, drug	OTSSP167 (OTS)	Cayman Chemical	Cat#16873; CAS:1431698-10-0	
Chemical compound, drug	1NM-PP1	Sigma-Aldrich	Cat#529581; CAS:221244-14-0	
Chemical compound, drug	4EGI-1	Cayman Chemical	Cat#15362; CAS:315706-13-9	
Chemical compound, drug	Cycloheximide (CHX)	Cayman Chemical	Cat#14126; CAS:66-81-9	
Chemical compound, drug	Defactinib	Cayman Chemical	Cat#17737; CAS:1073154-85-4	
Chemical compound, drug	PF-3758309	Cayman Chemical	Cat#19186; CAS:898044-15-0	
Chemical compound, drug	Nintedanib	Cayman Chemical	Cat#11022; CAS:656247-17-5	
Chemical compound, drug	SNS-032	Cayman Chemical	Cat#17904; CAS:345627-80-7	
Chemical compound, drug	Tozasertib	Cayman Chemical	Cat#13600; CAS:639089-54-6	Alternative Names : MK 0457, VX 680
Chemical compound, drug	GSK 626616	R and D Systems	Cat#6638; CAS:1025821-33-3	
Chemical compound, drug	EIPA	Sigma-Aldrich	Cat#A3085; CAS:1154-25-2	
Chemical compound, drug	(-)-Blebbistatin	Sigma-Aldrich	Cat#B0560; CAS:856925-71-8	
Software, algorithm	MATLAB R2014b	MathWorks		Used to analyze the SMR raw data and generate data plots.
Software, algorithm	OriginPro 2019	OriginLab		Used to perform statistical analyses and generate data plots.
Software, algorithm	Mass accumulation rate analysis code	This paper		Used to analyze the SMR data. Code can be found attached to this manuscript.

### Cell lines, primary cells and culture conditions

L1210 cells were obtained directly from ATCC, with the exception of L1210 cells shown in Figure 2—figure supplement 2b, which were obtained from ECACC. L1210 cells expressing the FUCCI cell cycle sensor were generated in a previous study (Son et al., 2012) using ATCC originating cells. The BaF3 cells were originally obtained from RIKEN BioResource Center and engineered to express BCR-ABL in a previous study (Stevens et al., 2016). S-HeLa cell line was a gracious gift from Dr Elias. FL5.12 cell line was a gracious gift from Dr Vander Heiden. All DT40 cell experiments were carried out using DT40 CDK1as cell line, which was a gracious gift from Dr Samejima and Dr Earnshaw. The DT40 CDK1as cells have had their CDK1 replaced with *Xenopus laevis* CDK1 with a F80G mutation, as detailed in Gibcus et al. (2018), to sensitize the CDK1 to inhibition by 1NM-PP1. Note that the CDK1as cell line is protected by MTA and the rights to this cell line belong to Prof Earnshaw and the University of Edinburgh. All cell lines tested negative for mycoplasma. Cell line identity was validated by vendors and the identity of L1210 cells from ATCC was further validated using RNA-seq data.

The L1210 cells basic experimental and culture conditions were in 2 mM L-glutamine and 11 mM glucose containing RPMI (Thermo Fisher Scientific, #11835030) supplemented with 10% FBS (Sigma-Aldrich), 1 mM sodium pyruvate (Thermo Fisher Scientific), 20 mM HEPES (Thermo Fisher Scientific) and antibiotic/antimycotic (Thermo Fisher Scientific). In Figure 2—figure supplement 2a, where L1210 cells were grown under different nutrient conditions, the media was kept otherwise identical except for 4% FBS and indicated concentrations of glucose (Sigma-Aldrich) or galactose (Sigma-Aldrich). The L1210 experimental conditions also included 10 nM TMRE in the media, which did not affect cell growth.

BaF3, FL5.12 and DT40 CDK1as cells were grown in media identical to L1210 cells, with the exceptions that FL5.12 cell media was supplemented with 10 ng/ml IL-3 (R and D Systems) and DT40 cell media was supplemented with 3% chicken serum (Sigma-Aldrich). S-HeLa cells were grown in DMEM (Thermo Fisher Scientific) containing 10% FBS (Sigma-Aldrich), 1 mM sodium pyruvate (Thermo Fisher Scientific), 20 mM HEPES (Thermo Fisher Scientific) and antibiotic/antimycotic (Thermo Fisher Scientific).

Human peripheral blood mononuclear cells (PBMCs), including T-cells, were isolated from unpurified buffy coat (Research Blood Components) and activated as described previously (Cermak et al., 2016). Briefly, PBMCs isolation was carried out using Ficoll-Paque Plus density gradient (GE) according to the manufacturer’s recommendations. After isolation of the PBMC layer, the cells were subjected to red blood cell lysis using ACK lysis buffer (Thermo Fisher Scientific). The PBMCs were then washed three times and either frozen or used for isolation of specific T-cell subsets. The T-cell data shown in this paper is derived from both frozen and non-frozen samples obtained from two independent blood samples. Primary T-cells were isolated using Naive CD8 +or CD3+T Cell Isolation Kits (Miltenyi Biotec) according to supplier’s instructions. The primary T-cells were activated on an anti-human CD3 (BioLegend) coated cell culture plate in identical to L1210 culture media, with the exception that the media was supplemented with 10 µM 2-mercaptoethanol, 100 U/ml IL-2 (R and D Systems) and 2 µg/ml anti-human CD28 (BioLegend). The same media was used for SMR experiments. After activation, cells were left undisturbed for 24 hr before SMR experiments, and the activation was validated by monitoring cell number and volume changes using Coulter Counter (Beckman Coulter).

The shRNA expressing L1210 cells were generated as in Kang et al. (2019). L1210 cells (obtained from ATCC) were transfected using mammalian shRNA knockdown lentiviral vectors obtained from VectorBuilder Inc Each construct contained an shRNA sequence under a U6 promoter, as well as EGFP and Puro linked by T2A for selection. Full construct details can be found online at VectorBuilder.com (Vector IDs: VB190401-1106ebw, VB190401-1107bty and VB190401-1108sfm)

Control shRNA target sequence: CCTAAGGTTAAGTCGCCCTCG

4E-BP1 shRNA #1 target sequence: ATTATCTATGACCGGAAATTT (location: 233–253, CDS)

4E-BP1 shRNA #2 target sequence: CCAGTGTTTATGGTGTGATTT (location: 912–932, 3’UTR)

Transfection was carried out using 4 rounds of spinoculation. In each round, 1.5 × 10^5^ L1210 cells were mixed with 10 µg/ml Polybrene (EMD Millipore) and approximately 1 × 10^6^ transducing units of lentivirus. The mixture was centrifuged at 800 g for 60 min at 25°C, and the cells were moved to normal culture media. This procedure was repeated every 12 hr for a total of 4 times. 24 hr after the last round of transfection selection was started using 10 µg/ml puromycin (Sigma-Aldrich). After 5 days of selection the shRNA and EGFP expressing subpopulation was sorted out using BD FACS Aria. shRNA knockdown efficiency was validated by immunostaining 4E-BP1 and quantifying the staining levels using FACS (antibody staining details are below, Figure 6—figure supplement 4a,b).

### SMR device setup and experimental details

The SMR chips were fabricated as previously described (Cermak et al., 2016; Son et al., 2015a; Son et al., 2012) by CEA-LETI, Grenoble, France. The device setup and experimental details were similar to those described previously (Cermak et al., 2016; Son et al., 2015a; Son et al., 2012). Briefly, a piezo-ceramic placed under the device vibrates the cantilever in its second flexural bending mode resonant frequency, which is typically ~1.1 MHz. The resonant frequency was monitored using piezo-resisters embedded at the base of the cantilever. A digital control platform was used to actuate the cantilever in a direct feedback mode, where an actuating signal is generated by amplifying and delaying the detected motion signal from the cantilever. Utilizing the feedback mode with a data rate of ~3000 Hz, our SMR measurement bandwidth was fixed to ~1500 Hz, which was adequate to capture fast modulating frequency signal resulting from a cell transit through the cantilever.

SMR device was operated at a fixed temperature of 37°C, by mounting the SMR chip on a copper clamp that was connected to a circulating, temperature-controlled water bath (Julabo). Fluid flow was controlled by pressure difference across the SMR input ports. Each input port was connected to a reservoir of normal cell culture media that was pressurized with 5% CO_2_, 21% O_2_ (Airgas) to maintain stable pH. The amount of pressure applied to each vial were controlled using electronic pressure regulators (Proportion Air QPV1) and the applied pressure difference across the channel was set to achieve a typical flow rate of ~2 nL/s to minimize the shear stress of cells growing within the SMR. This resulted in a typical ~300 ms transit time through the cantilever. Both the absolute pressure and flow direction were controlled using a custom software (LabVIEW 2012 and LabVIEW 2016). The software controls the pressure levels, and consequently the flow direction and speed, in real-time, and is automated to quickly respond to a change in resonant frequency signal. For example, a set of pressures are applied to flow a cell through the cantilever (Figure 1—figure supplement 1a, left #1). The resonant frequency change caused by cell transit through the cantilever automatically stops the flow (Figure 1—figure supplement 1a, left #2). Flow is maintained at zero for desired amount of time (~50 s), after which the pressures are changed to reverse the flow direction (Figure 1—figure supplement 1a, left #3). See Figure 1—figure supplement 1 for the detailed steps of the fluid control and consequent cell movement.

For monitoring morphology changes during mitosis and measuring FUCCI cell cycle reporter intensity, we utilized an on-chip imaging system described previously (Son et al., 2012). Briefly, a modular Nikon microscope equipped with a Nikon LU plan ELWD 50x/0.55 objective, a Lumencor Spectra X light engine and an 8 mm Voltage Output Type photomultiplier tube (Edmund Optics) or a monochrome camera (BFS-U3-13Y3M-C, FLIR). As a cell passed through the SMR cantilever, the change in the resonance frequency was used as a trigger to turn on illumination and measure the FUCCI reporter fluorescence. On-chip cell imaging was done as in Calistri et al. (2018).

### Data acquisition and processing

The motion of the cantilever and thus the resonant frequency of the SMR was measured by a digital control platform described previously (Cermak et al., 2016). The measured signal in digital platform was fed into custom LabVIEW code that records the signal while cell is in transit. Recorded frequency was then post-processed by custom MATLAB code, as described previously (Cermak et al., 2016). Briefly, the code locates two local minima in frequency peaks, fits a fourth order polynomial to the raw data, and the minimum resonance frequency values are extracted from the fittings. The average of these two resonance frequency minima measured in Hz was then transformed in to picograms by calibrating the measurements using monodisperse polystyrene beads (Thermo Fisher Scientific, Duke Standards) with a known buoyant mass. No frequency peak exclusion was performed, except in an extremely rare event where two daughter cells separate inside the cantilever during transit.

### Assigning cell cycle transitions to buoyant mass traces

We identified three distinct cell cycle transition points: mitotic entry (i.e. G2/M transition), metaphase-to-anaphase transition (i.e. M/A transition) and daughter cell abscission (Figure 1—figure supplement 2). We defined M-phase as the sum of mitosis and cytokinesis, starting at mitotic entry and ending at daughter cell abscission. We identified metaphase-to-anaphase transition using the mAG-Geminin signal of the FUCCI cell cycle reporter, which is degraded at metaphase-to-anaphase transition (Figure 1—figure supplement 2c), and using on-chip brightfield imaging, where we identified the metaphase-to-anaphase transition as the last timepoint of cell being round. These signals always coincided with two biophysical signals measured by SMR, a drop in node deviation signal (an acoustic signal corresponding to cell shape and stiffness) and a momentary reduction in buoyant mass trace (Figure 1—figure supplement 2b), both partly due to cell elongation in anaphase (Kang et al., 2019). These elongation dependent signals are applicable to all cell types studied here and this was validated by on-chip imaging, allowing us to designate metaphase-to-anaphase transition for all cells.

The daughter abscission was assigned for all cells based on the approximately 50% loss of buoyant mass within 2 min. Cytokinesis was defined as the time between metaphase-to-anaphase transition and daughter cell abscission. G1 was defined to start immediately following cell abscission.

Detection of mitotic entry (G2/M transition) for L1210 cells was carried out using biophysical parameters. First, we have previously shown that node deviation starts to decrease following mitotic entry (Kang et al., 2019) (Figure 1—figure supplement 2b), allowing us to locate mitotic entry for L1210 traces. The timing of the assigned mitotic entry (30 min prior to metaphase-to-anaphase transition) also matched with previously analyzed mitotic entry point based on single-cell volume measurements (Son et al., 2015a; Zlotek-Zlotkiewicz et al., 2015). Similar node deviation based assignment of approximate mitotic entry was also done for other cell types, whenever node deviation changes were observable. We also estimated the approximate mitotic entry by comparing the whole cell cycle duration to that of L1210 cells. When cell cycle durations were similar, we utilized the same timing of mitotic entry (30 min prior to metaphase-to-anaphase transition) for the other cell types, which was also consistent with the timing of mitotic entry observed by node deviation measurements. While this is an approximation, it is consistent with the notion that the duration of mitosis does not vary drastically cell-to-cell (Araujo et al., 2016). For chemical perturbations that prolonged early mitosis, the approximate mitotic entry is separately indicated in the figures (for example, arrows in Figure 6d).

Approximate duration of L1210 cell metaphase was assigned using the G2/M and metaphase-to-anaphase transitions together with previous characterization of the relative durations of different mitotic stages in L1210 cells (Son et al., 2015a). The duration of cell elongation (i.e. anaphase) was quantified for L1210 cells previously (12 min) (Kang et al., 2019) and the duration was approximated for other cell types using on-chip imaging.

### Analyzing mass accumulation rate (MAR) and MAR/mass

To quantify average MAR/mass of for late G2, early mitosis, cytokinesis and newborn G1, as seen for example in Figure 7a,a single linear fit was made to the buoyant mass traces for each indicated cell cycle stage and the slope of the fit represents the MAR (Figure 2—figure supplement 1a). To minimize the error in the length of fitted segment, data points were linearly interpolated from the buoyant mass trace to accurately pinpoint the beginning and the end of the fitted segments. Then, each slope of the linear fits (MAR) was divided by the average mass during the fitting period to obtain MAR/mass.

To quantify MAR/mass dynamics within mitotic stages, as seen for example in Figure 2a, we quantified the slope and average mass during each 10 min segments in the buoyant mass traces (Figure 2—figure supplement 1b). The 10 min segments were separated by 5 min. The 10 min segments were separately fitted a linear line, and each slope of the fitted line represents MAR (Figure 2—figure supplement 1c). Then MAR of each 10 min segment was divided by the average mass within that 10 min segment to calculate MAR/mass (Figure 2—figure supplement 1d). To minimize the error in the length of fitted segment, data points were linearly interpolated from the buoyant mass trace to increase number of data points. The MAR/mass dynamics shown in Figure 5; Figure 5—figure supplement 1; Figure 5—figure supplement 2 were processed similarly but the length of each segment and separation between each segment were reduced to 4 min and 2 min, respectively. When showing MAR/mass dynamics of individual cells (Figure 2d; Figure 2—figure supplement 1c,d; Figure 5—figure supplement 2a), buoyant mass traces were filtered using a moving average covering a 10 min window of data.

To correct for the cell elongation induced bias in buoyant mass measurement during cytokinesis, we performed a data correction as shown before (Kang et al., 2019). Briefly, the change in cell mass distribution (cell elongation) reduces the resonance frequency shift of the SMR, in a manner that is dependent on cell geometry. Using on-chip imaging, cell mass and volume information, we estimated the cell geometry to be i) spherical before elongation, ii) overlapping spheres during anaphase, and iii) spherical doublets after elongation is over (Figure 1—figure supplement 1d,e). With this information we can calculate the estimated extent of the measurement bias. The details can be found in the (Kang et al., 2019) and in the MATLAB code attached to the supplements. The calculated extent of the measurement bias during L1210 cell cytokinesis can be seen in Figure 1—figure supplement 1d and in Figure 2—figure supplement 1d.

### Chemical perturbations

All SMR experiments with chemical perturbations of cell growth or cell cycle, apart from serial SMR experiments, were carried out by diluting the chemicals directly in to the media within SMR. Untreated cells were then loaded to the SMR for growth monitoring, so that in a typical experiment the cell was exposed to the chemical for at least 2 hr before entering mitosis. Experiments with chemical inhibitors only lasted through one cell division, so that exposure to the chemicals was always started in interphase, approximately 1–4 hr prior to mitosis. Thus, the n indicated for chemical treatments always reflects separate experiments. Control experiments were always carried out between experiments with chemical perturbations to assure that control cell growth rates were reproducible. Note that the control L1210 cell data is accumulated over several years and the growth behavior was reproducible also between different SMR devices (Figure 2—figure supplement 2c).

For serial SMR experiments where cells were arrested to mitosis using STLC, cells were loaded in to the serial SMR in normal culture media immediately after STLC wash off. For serial SMR experiments where cells were treated with RO-3306, cells were loaded in to the serial SMR in 1 µM RO-3306 containing culture media immediately after release from G2 arrest. In Figure 4c, mitotic arrests were obtained by treating unsynchronized L1210 cells with 5 µM STLC, 1 µg/ml Nocodazole, 2 µM MG132 or 20 µM proTAME.

Importantly, we observed that RO-3306 stock stored in −20°C in DMSO was not stable over several months and we therefore carried out all experiments using RO-3306 that was prepared within 1 week of the experiment.

### Cell cycle synchronizations

L1210 cells were synchronized to G2 using a double thymidine block followed by a RO3306 mediated G2 arrest. L1210 cells in confluency of 4*10^5^ cells were first treated with 2 mM Thymidine for 15 hr, then washed with PBS and moved to normal culture conditions to release from G1/S arrest. After 6 hr, 2 mM Thymidine was added for 6 hr. Cells were again washed with PBS and moved to normal culture conditions for 3 hr, after which 5 µM RO-3306 was added. 7 hr later cells were arrested in G2 (Figure 3a). Cells were then washed with PBS and moved to normal culture conditions (unless otherwise stated) to allow cells to uniformly progress through mitosis. Note while most cells enter mitosis soon after release from G2 arrest, some cells fail to exit G2. The release from G2 arrest was considered as time zero in protein synthesis, serial SMR and proliferation experiments.

When cells were temporarily arrested in mitosis (Figure 7c,d,f), cells were first synchronized to G2, then released in to 5 µM STLC. 4 hr later the cells were washed twice with media and replaced in to normal cell culture conditions. The release from STLC mediated mitotic arrest was considered as time zero in protein synthesis, serial SMR and proliferation experiments.

### Protein synthesis rate sample preparation

Protein synthesis rates were quantified using the Click-iT Plus OPP Alexa Fluor 594 Protein Synthesis Assay Kit (Thermo Fisher Scientific) together with antibody staining specific for different mitotic stages. For each replicate approximately 3*10^6^ cells were treated with 20 µM OPP for 10 min under normal culture conditions. The OPP accumulation was stopped by mixing the cells with ice cold PBS, after which the cells were quickly washed with ice cold PBS and then fixed with formaldehyde for 10 min at room temperature. To reduce the number of cell doublets fixed together, the fixation was carried out by shaking the cells on vortex in PBS and slowly adding a corresponding volume of 8% paraformaldehyde to reach a final paraformaldehyde concentration of 4%. Fixative was washed away with PBS and the cells were permeabilized using 0.5% Triton X-1000 in PBS for 10 min at room temperature. The permeabilization solution was washed away with PBS and cells were incubated in 5% BSA in PBS for 30 min to block non-specific antibody binding.

Mitotic cells were separated from interphase cells using p-Histone H3 monoclonal antibody (S10, D2C8, conjugated to Alexa 647, Cell Signaling Technology, #3458S; or S10, D2C8, conjugated to Alexa 488, Cell Signaling Technology, #3465S). For separation of mitosis in to early and late mitosis, Cyclin B1 (V152, conjugated to Alexa 488, Cell Signaling Technology, #4112S) and Cyclin A (H-3, conjugated to FITC, Santa Cruz Biotechnology, #sc-271645 FITC) monoclonal antibodies were used. All antibody labeling steps were carried out o/n in 4°C in 5% BSA containing PBS. All antibodies were used at the concentration recommended by supplier. Antibodies were washed away using 5% BSA in PBS.

The OPP Click-IT reaction was carried out according to manufacturer’s (Thermo Fisher Scientific) instructions. After OPP conjugation with Alexa Fluor 594 fluorophore, the cells were washed twice with PBS and DNA was stained for 30 min in RT with 1:2000 dilution of NuclearMask Blue (#H10325, Thermo Fisher Scientific). Finally, the cells were washed three times with PBS, mixed in to PBS supplemented with 1% BSA and put on ice until FACS analysis.

### Protein content and phosphorylation level sample preparation

To analyze the levels of total 4E-BP1 or phosphorylated S6RP and 4E-BP1, unperturbed cells were prepared using same fixation, permeabilization and blocking protocol as for protein synthesis assays. The cells were then incubated o/n in 4°C with 4E-BP1 monoclonal antibody (53H11, Cell Signaling Technology, #34470), p-4E-BP1 monoclonal antibody (Thr37/46, 236B4, conjugated to PE, Cell Signaling Technology, #7547S), p-S6RP monoclonal antibody (S235/236, D57.2.2E, conjugated to Alexa 647, Cell Signaling Technology, #4851S) or isotype specific controls (Rabbit mAb IgG, conjugated to PE or Alexa 647, Cell Signaling Technology, #5742S) in PBS solution containing 5% BSA. All antibodies were used at the concentration recommended by supplier. For analysis of total 4E-BP1 levels, cells were washed and treated with 2 µg/ml secondary antibody (Goat anti-Rabbit IgG secondary antibody conjugated to Alexa Fluor 568, #A-11011, Thermo Fisher Scientific) for 2 hr in RT. Antibodies were washed away using 5% BSA in PBS and the cells were stained for p-Histone H3 (S10) and DNA, as in protein synthesis assays. Finally, the cells were washed three times with PBS, mixed in to PBS supplemented with 1% BSA and put on ice until FACS analysis.

### Flow cytometry

FACS-based quantifications were done using BD Biosciences flow cytometer LSR II HTS with excitation lasers at 355 nm, 488 nm, 561 nm and 640 nm, and emission filters at 450/50, 530/30, 585/15 and 660/20. See (Figure 3a,c; Figure 3—figure supplement 1a) for DNA and antibody labeling that were used to separate cell cycle stages. At least 20,000 cells were analyzed for each replicate so that each analyzed subpopulation contained at least 250 cells, and typically over 1000 cells.

### Microscopy

For validation of OPP staining, L1210 cells were plated on coverslips coated with 0.1% poly-L-lysine, and prepared using same fixation, permeabilization, blocking, antibody labeling and DNA staining protocol as for protein synthesis assays. After all staining procedures were done, the cells were mounted on to microscopy slides in Vectashield mounting medium (Vector Laboratories).

For examination of phosphorylated 4E-BP1, L1210 cells were plated on coverslips coated with 0.1% poly-L-lysine, and prepared using same fixation, permeabilization and blocking protocol as for protein synthesis assays. The cells were then labeled o/n in 4°C with p-4E-BP1 monoclonal antibody (Thr37/46, 4EB1T37T46-A5, #MA5-27999, Thermo Fisher Scientific). The following day the cells were washed three times with PBS and then treated with 2 µg/ml secondary antibody (Goat anti-Rabbit IgG secondary antibody conjugated to Alexa Fluor 568, #A-11011, Thermo Fisher Scientific) for 2 hr, before an o/n labelling in 4°C with α-Tubulin monoclonal antibody (11H10, conjugated to Alexa Fluor 488, #5063, Cell Signaling Technology). The following day the cells were washed three times with PBS and DNA was stained for 30 min in RT with 1:2000 dilution of NuclearMask Blue (#H10325, Thermo Fisher Scientific). The cells were washed three times with PBS and mounted on to microscopy slides in Vectashield mounting medium (Vector Laboratories). OPP staining and 4E-BP1 phosphorylation levels were imaged using DeltaVision wide-field deconvolution microscope using standard filters (DAPI, FITC, TRITC) and 100 × objective. After deconvolution using SoftWoRx 7.0.0 software, approximately 3 µm thick section from the middle of z-slices was merged into a maximum intensity image for visualization.

Cell proliferation rate was analyzed by imaging the cells every 1 hr using IncuCyte live cell analysis imaging system by Sartorius. The relative cell count was then assessed from the phase images by analyzing the relative confluency in each sample. These values were normalized to the value at start and hourly average values were plotted together with the standard error of mean (SEM). Representative images displaying the duration of daughter cell separation (Figure 2c,e) were obtained in a parallel experiment using IncuCyte live cell analysis imaging system by Sartorius with 20X objective or using on-chip imaging in the SMR with the imaging setup detailed in the section ‘*SMR device setup and experimental details*’.

### Lambda protein phosphatase treatment

To verify the phospho-specificity of the p-4E-BP1 antibodies, cells were prepared using same fixation, permeabilization and blocking protocol as detailed above. The cells were first treated with p-Histone H3 antibody to label mitotic cells and to protect p-Histone H3 sites from Lambda phosphatase, after which the phosphatase treatment and DNA staining were carried out. The cells were then treated with 10,000 units/ml of Lambda protein phosphatase in 1X NEBuffer for protein MetalloPhosphatases that contained 1 mM MnCl_2_ for 12 hr in 30°C, after which cells were washed twice with PBS. The Lambda phosphatase and the treatment buffer were obtained from New England BioLabs (#P0753S). Finally, DNA was stained as detailed above and the cells were analyzed using flow cytometer.

### Combining and normalizing data

Total mass accumulation for each cell was calculated from the mass accumulation traces, by assuming that the birth size of the cell was exactly half of the abscission size. Mass accumulation during mitosis (between G2/M transition and metaphase-to-anaphase transition) and during cytokinesis (between metaphase-to-anaphase transition and daughter cell abscission) was then normalized to the calculated total mass accumulation for each cell. MAR/mass traces (i.e. MAR/mass vs time) from each single cell were aligned to metaphase-to-anaphase transition. We then linearly interpolated data points from each MAR/mass trace, consequently making the total number of timepoints for each cell to be 100. Mean and SD were calculated for each timepoint and plotted as a function of approximate mitotic entry (30 min before metaphase-to-anaphase transition for most cell types). In Figure 4, where the drug-induced mitotic arrests inhibit us from aligning the data to metaphase-to-anaphase transition, all data was aligned to approximate mitotic entry using node deviation and MAR/mass signals. In addition, in Figure 4d, the MAR/mass traces were smoothed with a moving average filter of length 3. Data smoothing was not done for any other datasets. In Figure 7a,b, average MAR/mass for each indicated cell cycle stage (late G2, early mitosis, cytokinesis and early G1) was normalized to control values within that cell cycle stage.

### Statistical information

All statistical tests carried out, as well as descriptions of error bars and numbers of replicates, are detailed in the figure legends. All t-tests were two tailed. No replicates were excluded, except for image analysis, when cells could not be analyzed, as detailed above. No power analysis was used. Sample size was kept at or above three independent replicates, with exact sample size depending on the experimental setup. Many of the experimental approaches required time-sensitive sample processing, which limited the maximum sample size. In all FACS-based assays, the replicate number refers to independent cell cultures. In all SMR-based assays, the replicate number refers to independent cells measured through mitosis. In SMR-based assays, control samples were grown for multiple generations yielding several replicates in one experiment, whereas drug treated samples were only grown through one division so that each drug treated replicate represents a separate experiment. Experiments were repeated at least three times on separate days, unless otherwise stated in the figure legends.

The increase in L1210 cell MAR/mass from G2 to early mitosis was quantified by comparing the highest MAR/mass values observed in G2 and in early mitosis for each cell separately. The statistical comparison between these two groups was carried out by two-tailed Student’s t-test.

Statistical tests were carried out using OriginPro 2019 software. The analysis of buoyant mass traces and the analysis of images was carried out using MATLAB R2014b. Visualization of microscopy images was carried out using ImageJ. All figures were compiled using Adobe Illustrator CC 2018.

### Data and data analysis code availability

All L1210 control buoyant mass measurement shown in Figure 1 and used for quantification of MAR/mass in Figure 2a can be found in Figure 1—source data 1. This data has not been corrected for the cell elongation. Data analysis code for correcting the cell elongation bias and obtaining MAR from the buoyant mass traces can be found attached to this manuscript.

## Data Availability

All L1210 control buoyant mass measurement around M-phase, which were used for quantification of mitotic growth (Figure 1), MAR/mass dynamics (Figure 2), can be found in Figure 1-source data 1.

## Additional files

Source code 1. Mass accumulation rate analysis code.

Transparent reporting form
